# Age‐Associated Transcriptomic and Epigenetic Alterations in Mouse Hippocampus

**DOI:** 10.1111/acel.70233

**Published:** 2025-09-28

**Authors:** Merve Bilgic, Rinka Obata, Vlada‐Iuliana Panfil, Ziying Zhu, Mai Saeki, Yukiko Gotoh, Yusuke Kishi

**Affiliations:** ^1^ Laboratory of Molecular Neurobiology, Institute for Quantitative Biosciences The University of Tokyo Tokyo Japan; ^2^ Laboratory of Molecular Biology, Graduate School of Pharmaceutical Sciences The University of Tokyo Tokyo Japan; ^3^ Laboratory of Molecular Neurobiology, Graduate School of Pharmaceutical Sciences The University of Tokyo Tokyo Japan; ^4^ Department of Cell and Molecular Biology Karolinska Institutet Stockholm Sweden; ^5^ International Research Center for Neurointelligence (WPI‐IRCN) The University of Tokyo Tokyo Japan; ^6^ Division of Aging Biology Research Institute for Science and Technology, Tokyo University of Science Chiba Japan

**Keywords:** aging, BACH2, chromatin accessibility, glia, hippocampus, neuron, single‐nucleus multiome, transcriptome

## Abstract

Aging represents a major risk for human neurodegenerative disorders, such as dementia and Alzheimer's disease, and is associated with a functional decline in neurons and impaired synaptic plasticity, leading to a gradual decline in memory. Previous research has identified molecular and functional changes associated with aging through transcriptomic studies and neuronal excitability measurements, while the role of chromatin‐level regulation in vulnerability to aging‐related diseases is not well understood. Moreover, the causal relationship between molecular alterations and aging‐associated decline in functions of different cell types remains poorly understood. Here, we systematically characterized gene regulatory networks in a cell type‐specific manner in the aging mouse hippocampus, a central brain region involved in learning and memory formation, by simultaneously profiling gene expression and chromatin accessibility at a single‐nucleus level. The analysis of multiome (RNA and ATAC) sequencing recapitulated the diversity of glial and neuronal cell types in the hippocampus and revealed transcriptomic and chromatin accessibility level changes in different cell types, among which oligodendrocytes and dentate gyrus (DG) neurons exhibited the most drastic changes. We found pronounced aging‐dependent chromatin‐level changes among neurons, especially for genes related to synaptic plasticity. Our data suggest that BACH2, a candidate transcription factor implicated in aging‐mediated functional decline of DG neurons, potentially regulates genes associated with synaptic plasticity, cell death, and inflammation during aging. Taken together, our single‐nucleus multiome analysis reveals potential cell type‐specific regulators involved in the aging of neurons and glial cells.

## Graphical abstract

Single‐nucleus multiome sequencing of hippocampus reveals aging‐dependent changes in gene expression and chromatin accessibility. Chromatin alterations in synaptic genes often occur without corresponding expression changes, suggesting a “susceptible” chromatin state. Age‐related regulatory shifts involving BACH2 and AP‐1 in dentate gyrus neurons may link chromatin and transcriptomic remodeling to brain aging.

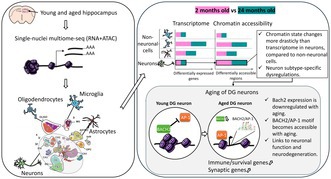

## 1 Introduction

The aging brain undergoes a series of transformations involving the hippocampus that have profound implications for memory and cognitive function. The hippocampus plays a pivotal role in memory formation and retrieval, and its functional decline with aging has implications in aging‐related disorders, including Alzheimer's disease (AD), due to impaired synaptic plasticity and intrinsic excitability (Nicholson et al. 2004; Lu et al. 2004; Oh et al. 2010). Understanding how aging‐associated changes occur in the hippocampus has been important in the field of neuroscience and medical sciences.

Previous research identified age‐associated transcriptomic and epigenetic alterations in the mammalian brain, implicating processes such as cellular senescence, inflammation, oxidative stress, decreased mitochondrial function, and protein processing (Guo et al. 2022; Wang et al. 2022; Campisi et al. 2019; López‐Otín et al. 2013; Hauss‐Wegrzyniak et al. 2000; Murray and Lynch 1998; Allen et al. 2023; Lee et al. 2000; Blalock et al. 2003; Lu et al. 2004). Moreover, epigenetics contributes to cellular memory via the regulation of DNA, histone modifications, and chromatin structure, and its alteration during the aging process of many tissues is well known (Wang et al. 2022; Talens et al. 2012; Han and Brunet 2012; Maybury‐Lewis et al. 2021; Ucar et al. 2017; Ernest Palomer et al. 2016). For example, the inhibition of histone deacetylase (HDAC) enhanced hippocampus‐dependent memory and synaptic plasticity (Vecsey et al. 2007; Guan et al. 2009), although other studies showed no difference (Harman and Martín 2020; Sewal et al. 2015). Manipulation of the DNA methyltransferases (DNMT) affected the memory formation of aged mice (Liu et al. 2011; Oliveira et al. 2012). However, the epigenetic alterations during hippocampal aging at the genome‐wide level remain unknown, while alterations in some specific gene loci were reported (Ernest Palomer et al. 2016).

Nevertheless, aging is a gradual process, marked by cumulative effects of cellular damage that occur asynchronously across individuals, tissues, and cells (Buckley et al. 2022). Previous research revealed that aging has different effects on different types of neurons in structurally and functionally distinct hippocampal regions, including the dentate gyrus (DG), Cornus Ammonis‐1 (CA1), CA2, CA3, and subiculum (Flood, Buell, et al. 1987; Flood 1991; Flood, Guarnaccia, and Coleman 1987; Hanks and Flood 1991; Maziar et al. 2023). These observations highlight the different vulnerabilities of neurons to the aging process and neurodegenerative diseases. Single‐cell omics studies emerged as a powerful tool to profile aging‐dependent transcriptomic and epigenetic changes in healthy and diseased brains at a single‐cell level (Ogrodnik et al. 2021; Ximerakis et al. 2019; Luo et al. 2023, 2022; Shao et al. 2021; Zhang et al. 2022; Hajdarovic et al. 2022; Shi et al. 2018). Many studies identified the specific molecular state of microglia, immune residents in the brain, in normal aging and in AD conditions in mice and humans (Li et al. 2023; Xiong et al. 2023; Sun et al. 2023; Lopes et al. 2022). Others found a regional specificity in aging–dependent changes of gene expression in glial cells, non‐neuronal cell types supporting neuronal development and function (Soreq et al. 2017; Astillero‐Lopez et al. 2022). Excitatory and inhibitory neurons in the hippocampus of patients with AD exhibited region‐specific transcriptomic changes (Luo et al. 2023; Mathys et al. 2023). In addition to single‐cell transcriptomic analysis, single‐nucleus ATAC‐sequencing (snATAC‐seq) allowed researchers to generate an atlas of cis‐regulatory DNA elements (CREs) of different cell types in the adult mouse brain (Li et al. 2021; Zu et al. 2023; Yao et al. 2021).

Despite these advancements, how cell type–specific alterations of CREs during hippocampal aging and their contribution to aging‐related vulnerabilities of different neuronal subtypes in the hippocampus remains less understood. To address these limitations, we investigated the dynamic alterations in gene expression and chromatin accessibility in the hippocampus by employing single‐nucleus multiome sequencing of RNA and ATAC libraries from the same nuclei. This method allows us to obtain an improved sensitivity and proper specificity of CRE‐gene expression links compared with computational integration of separately performed snRNA‐seq and snATAC‐seq assays (Xie et al. 2023). We assessed cell type–specific age‐dependent changes by comparing young and aged cells for each cell type. Our results revealed that glial cells exhibited more pronounced transcriptomic changes compared with neurons. Among neurons, DG neurons were most affected by aging, followed by CA1 and subiculum. Notably, rather than gene expression, most of the synaptic plasticity and other neuronal function–related genes were altered at the chromatin level. Motif enrichment analyses highlighted the BACH2 transcriptional repressor as a potential regulator of the aging process in DG neurons, shaping the epigenetic and transcriptomic landscape of synaptic and survival genes.

## 2 Results

### 2.1 Single‐Nucleus Profiling of Transcriptome and Chromatin Accessibility in the Aging Mouse Hippocampus

**FIGURE 1:**
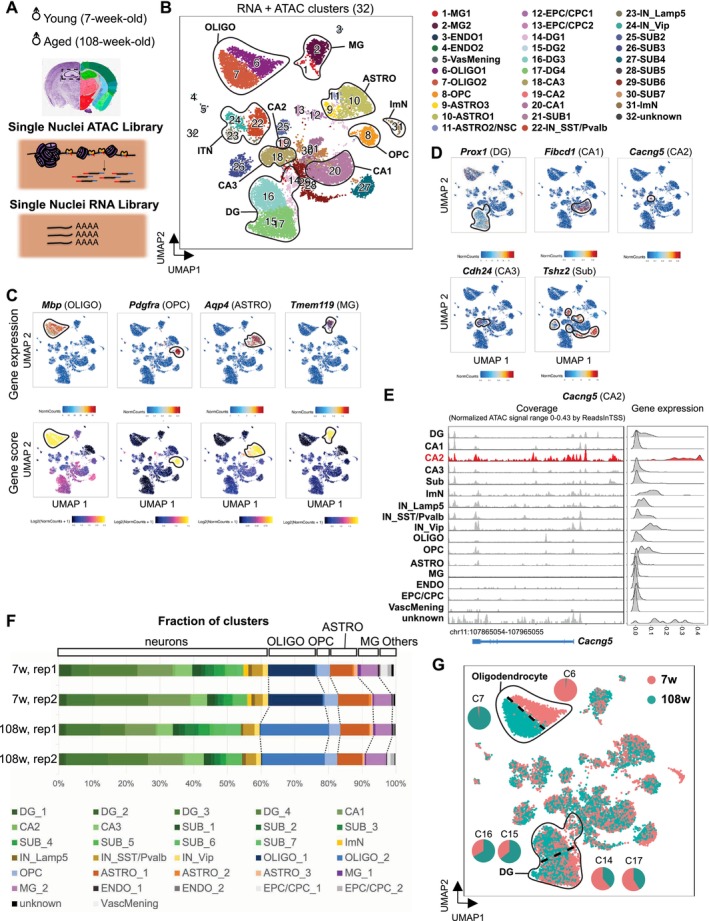
Single‐nucleus profiling of transcriptome and chromatin accessibility in the aging mouse hippocampus. (A) Scheme of experimental procedure for generating single‐nucleus ATAC‐seq and single‐nucleus RNA‐seq libraries using two biological replicates of young (7‐week‐old) and aged (108‐week‐old) male mice. Nissl and anatomical annotations were taken from the Allen Reference Atlas—Mouse Brain, atlas.brain‐map.org. (B) Visualization of clusters on UMAP generated by combining two modalities (RNA and ATAC). Cells are colored by clusters. (C, D) Expression patterns of glial cell markers (C) and neuronal markers (D) are shown on UMAP. Clusters that strongly expressed these markers on the basis of heatmap intensity were encircled to highlight corresponding cell types. (C) *Mbp*, *Pdgfra*, *Aqp4*, and *Tmem119* were used as representative markers for oligodendrocytes, oligodendrocyte precursors, astrocytes, and microglia, respectively. Gene expression (RNA assay, above) and gene score (ATAC assay, below) for these representative genes are shown. (D) Gene expression (RNA) for neuronal markers is shown on UMAP. *Prox1*, *Fibcd1*, *Cacng5*, *Cdh24*, and *Tshz2* were used to represent DG, CA1, CA2, CA3, and SUB neurons, respectively. (E) Genome browser track (left) and gene expression (right) for *Cacng5*, a representative marker of CA2 neurons. (F) Percentage of clusters in B in two replicates from young to aged samples is shown. (G) Visualization of cells on UMAP, colored by the age of sample collection. Age‐dependent separation of OLIGO and DG is highlighted. Among OLIGO, 98.2% of cluster 6 consists of aged cells, while 97.7% of cluster 7 consists of young cells. Among DG, 66.7% and 64.1% of clusters 15 and 16 represent aged cells, while 61.4% and 59% of clusters 14 and 17 represent young cells.

To understand the aging‐associated changes of gene regulatory programs in different cell types of the hippocampus, we profiled the transcriptome and chromatin accessibility of hippocampal cells isolated from two biological replicates each of 7‐week‐old (young) and 108‐week‐old (aged) male mice (Figure 1A). We took advantage of single‐cell multiomics to capture the cellular heterogeneity and minimize the variance of aging‐related profiles across samples by obtaining both RNA and ATAC libraries from the same single nuclei. This method allowed a direct comparison between the gene expression and chromatin state of genes and minimized sample variance by simultaneously mapping transcriptomic landscapes and chromatin accessibility within individual cells, hence enhancing the depth of our analysis. We filtered low‐quality cells and performed dimension reduction and clustering using ArchR software (Methods; Granja et al. 2021). The quality control filtering retained 15,480 nuclei (Figure S1A; Table S1; 3815 for young replicate 1; 3569 for young replicate 2; 3834 for aged replicate 1; 4262 for aged replicate 2). Peak calling by MACS2 identified 478,861 chromatin accessibility peaks merged from all four samples distributed across distal (35%), intronic (53%), promoter (6%), and exonic (6%) regions (Figure S1B; Table S1). Clustering was performed using two modalities (RNA and ATAC) in an unbiased manner and resulted in 32 entities that recapitulated major cell types including excitatory neurons (ExN) in DG, CA1, 2, 3, and subiculum (SUB), and interneurons (ITN) including somatostatin‐ or parvalbumin‐expressing interneurons (SST/Pvalb), Vip‐expressing interneurons (Vip), Lamp5‐expressing interneurons (Lamp5), astrocytes (ASTRO), immature neurons (ImN), oligodendrocytes (OLIGO), oligodendrocyte precursor cells (OPC), microglia (MG), and endothelial cells (ENDO) (Figure 1B). The clusters were annotated by the composition of RNA expression and chromatin accessibility score (“gene score”), a predictor of gene expression based on accessible peaks surrounding the gene, for key cell type markers (Figures 1C,D and S1C; Table S1). We validated our annotation for neuronal subtypes, clusters of excitatory neurons named DG (“*Prox1*”), CA1 (“*Fibcd1*”), CA2 (“*Cacng5*”), CA3 (“*Cdh24*”), and SUB (“*Tshz2*”) by visualizing RNA expression and chromatin accessibility of their representative markers (Figure 1D,E). In addition, a distinct subset of astrocytes with neural stem cell‐like properties was identified and shown to decline with age (Figure S1D–F; Table S1; Data S1). Importantly, multimodal integration of RNA and ATAC data further enhanced the resolution of neuronal subtypes compared to single‐modality analyses (Figure S2A,B; Data S1). Finally, clustering quality and accuracy of cell type annotations were confirmed using neighborhood purity and label transfer analyses (Figure S2C,D; Table S1; Data S1).

Our comprehensive profiling illuminated significant age‐dependent variations. Neuron populations dominated both young and aged samples by constituting over 60% of the samples due to a nuclear isolation method allowing the capture of viable neurons (Figure 1F; Lake et al. 2016). We noticed that OLIGO was increased overall in frequency with aging (31% in young and 40% in aged), as also observed in other datasets (Allen et al. 2023), and significantly separated in an age‐dependent manner on UMAP (Figure 1F,G); clusters 6 and 7 were young‐enriched and aged‐enriched, respectively. Similarly, aged and young DG neurons also tended to group together (Figure 1G; Table S1); clusters 14 and 17 were young‐enriched, and 15 and 16 were aged‐enriched.

Taken together, we generated single‐nucleus transcriptome and open chromatin profiles of young and aged hippocampus from the same nuclei where the combination of these two assays resulted in a greater resolution of clustering and reflected the age‐dependent separation of subtypes.

### 2.2 Cell Type–Specific Dynamics of Transcriptome and Epigenome During Hippocampal Aging

The aging‐dependent segregation of OLIGO and DG clusters on UMAP implied that these cells might undergo more pronounced changes in gene expression and chromatin state with aging compared to other cell types. To assess age‐related alterations specific to each cell type, we analyzed DEGs and DARs by comparing young and aged cells within each cell type (Methods). ASTRO2/NSC cells were excluded from this analysis due to their limited cell number in aged samples (see Data S1; Figure 1F).

**FIGURE 2:**
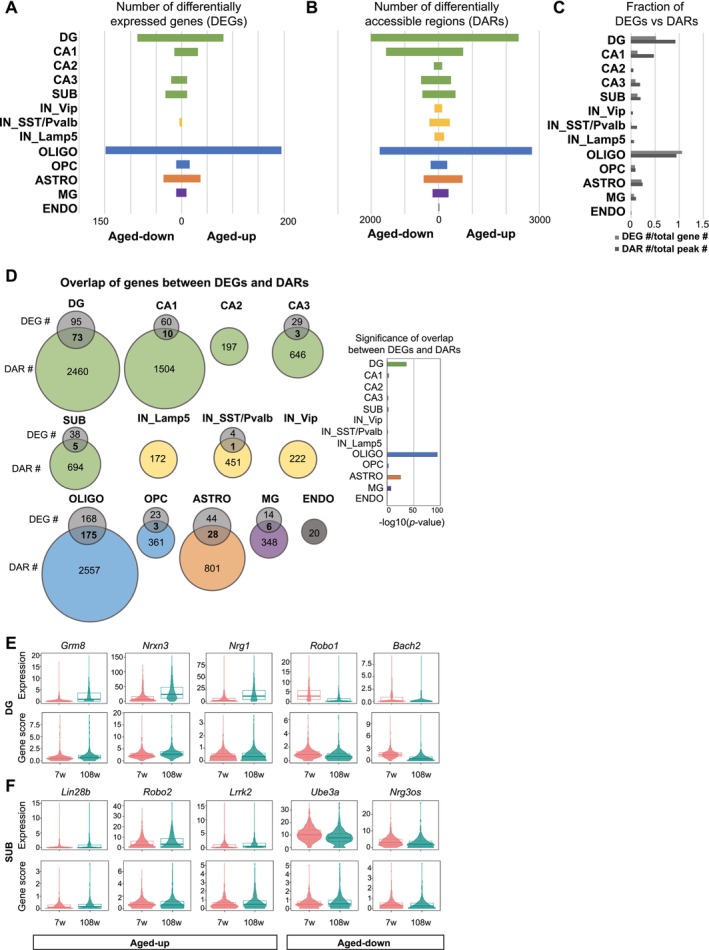
Cell type–specific transcriptome and epigenome dynamics across hippocampal aging. (A, B) Number of DEGs (A) and DARs (B) by each major cell type. The graph is centered at 0 counts and divided into right and left directions to represent aged‐upregulated (aged‐up) and aged‐downregulated (aged‐down) genes or peaks, respectively. The color code of bars is determined by each major cell type. (C) Bar plot showing the fraction of DEGs to total genes (light gray) and the fraction of DARs to total peaks (dark gray). (D) Venn diagrams showing the number of genes obtained in DEG analysis (RNA) and DAR analysis (ATAC). Overlap of genes between DEGs and DARs is highlighted in bold. Significance of overlap was quantified by Fisher's exact test and −log_10_ (*p*‐value) is plotted. *p*‐values calculated for each cell type are as follows: DG, 2.4e‐36; CA1, 1.5e‐03; CA2, 1; CA3, 0.026; SUB, 2.3e‐03; IN_Lamp5, 1; IN_SstPvalb, 0.068; IN_Vip, 1; Oligo, 6.9e‐95; OPC, 3e‐03; ASTRO, 5.1e‐32; MG, 5.6e‐08; ENDO, 1. (E, F) Violin plots for gene expression (RNA) and gene score (ATAC) for DG‐specific (E) or SUB‐specific (F) representative overlapping aging genes in (D).

We examined the aging–dependent transcriptomic changes in different neuron subtypes and in glial cells, including OLIGO (344 DEGs; adjusted *p*‐value < 0.05) and ASTRO (72 DEGs; adjusted *p*‐value < 0.05; Figure 2A; Table S2). Among excitatory neurons, DG neurons underwent the most pronounced transcriptomic changes with aging, followed by CA1, SUB, and then CA3 neurons (DG: 169; CA1: 46; CA2: 0; CA3: 32; SUB: 43 DEGs; adjusted *p*‐value < 0.05; Figure 2A; Table S2). At the chromatin level, neuron subtypes exhibited significant aging–associated changes, with a higher proportion of DARs compared to the total peaks detected than the proportion of DEGs to the total genes (Figure 2A–C). For example, the fraction of DARs in DG, CA1, and SST/Pvalb represented 0.92%, 0.48%, and 0.13% of total peaks, while the fraction of DEGs was 0.52%, 0.14%, and 0.02% among total genes, respectively (Figure 2C). By contrast, glial cells showed a more balanced number of changes in their RNA and open chromatin profiles, indicating that both their transcriptome and chromatin accessibility were affected to a similar degree by aging (Figure 2C). Notably, DG neurons and OLIGO exhibited the most drastic changes at both RNA and chromatin levels. Moreover, DEG analysis detected more aging–associated DEGs in non‐neuronal cells, including OLIGO and ASTRO, than in most neuronal subtypes, supporting previous observations (Allen et al. 2023).

The comparison of the fraction of annotated DARs altered during aging (aging DARs) revealed a notable enrichment of aging DARs in promoter regions in aged OLIGO and OPC compared to their young counterparts (Figure S3A; Table S2). In excitatory neurons (DG, CA1–3, SUB), there was a global decline in distal intergenic peaks with aging and an increase in peaks on intronic and promoter regions in those cells, suggesting a shift in the regulatory landscape of these cells. Neuron subtypes, including DG, CA1, CA2, CA3, and SUB, displayed a higher number of nearest genes of aging DARs compared to the number of detected aging DEGs (Figure 2D). This result indicated that neurons underwent more profound changes at the chromatin level compared to glial cells. Consistent with this, the nearest genes of DARs in CA1, CA2, CA3, and SUB neurons showed no or fewer overlaps with DEGs, while glial cells, such as OLIGO and ASTRO, had significant overlap (Figures 2D and 4). This discrepancy in neurons indicates alterations in chromatin regulation in neurons that do not directly translate to gene expression changes, suggesting a chromatin state that is susceptible to aging features.

To explore the aging‐dependent gene regulatory mechanisms in different cell types, we have examined cell type‐specific genes altered at both expression and chromatin levels. Notably, SUB neurons, a critical output structure of the hippocampus, have been relatively understudied in the context of aging. Our analysis identified specific dysregulation of synaptic genes within DG neurons, such as *Grm8*, *Nrxn3*, *Nrg1*, and *Robo1*, and immune‐responsive transcription regulators, such as *Bach2*, suggesting the involvement of both synaptic and inflammatory components in aging (Figures 2E and 5). In SUB neurons, *Lin28b*, *Robo2*, *Lrrk2*, *Ube3a*, and *Nrg3os* were among the dysregulated genes (Figure 2F). These genes play significant roles in various neuron functions, such as synaptic formation and axon guidance (Stolla et al. 2019; Blockus et al. 2021; Kuhlmann and Milnerwood 2020; Furusawa et al. 2023; Müller et al. 2018). Their dysregulation sheds light on potential molecular mechanisms that might explain the vulnerability of SUB neurons, such as age‐related decline in episodic memory (Chi et al. 2022). These findings highlight the specific molecular mechanisms potentially underlying aging‐related vulnerability of SUB and DG neurons.

To further examine the implication of AD risk genes among neuron subtypes with aging, we computed a module score using a set of genes altered during human AD progression based on a previous microarray analysis on the CA1 and CA3 regions (Table S2; Methods; Miller et al. 2013). Our analysis revealed that only DG and CA1 neurons exhibited significant changes with aging in the expression of genes downregulated or upregulated in AD, while other neuron subtypes did not show significant alterations (Figure S3B). Similarly, at the chromatin accessibility level, aging DARs showed significant alterations in DG, CA1, and CA2 (Figure S3B). This suggests that both transcriptomic and chromatin accessibility changes in these AD risk genes are prominent in DG, CA1, and CA2 neurons.

Taken together, these results highlight the differential impact of aging across cell types and suggest a preparatory chromatin state for genes associated with neuronal dysfunction during aging. This state may contribute to the increased susceptibility of neurons to age‐related stressors.

### 2.3 Dysregulation of Neuronal Genes in Glial Cells During Hippocampal Aging

**FIGURE 3:**
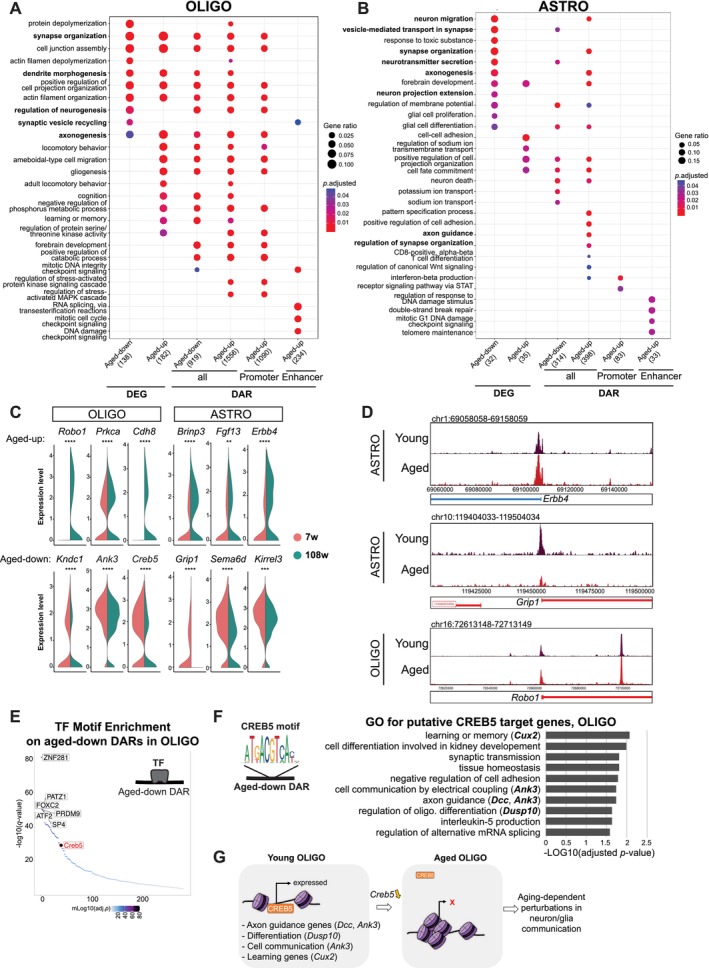
Dysregulation of neuronal genes in glial cells during hippocampal aging. (A, B) Gene ontology enrichment analysis of DEGs, genes located near DARs, including all DAR peaks, peaks on promoters, and enhancers, separately. (A) OLIGO. (B) ASTRO. (C) Violin plots for gene expression for synaptic function‐related genes in OLIGO and ASTRO. Adjusted *p*‐values obtained with Seurat's *FindMarkers* function with the MAST algorithm using a hurdle model as a statistical test are shown (ns, non‐significant; *Adjusted *p* < 0.05; **Adjusted *p* < 0.01; ***Adjusted *p* < 0.001; ****Adjusted *p* < 0.0001; Table S2). Adjusted *p*‐values calculated for each gene are as follows: *Robo1*, 1.21e‐71; *Prkca*, 2.74e‐10; *Cdh8*, 2.86e‐166; *Kndc1*, 1.27e‐16; *Ank3*, 9.44e‐19; *Creb5*, 1.87e‐44; *Brinp3*, 9.19e‐26; *Fgf13*, 0.007; *Erbb4*, 6.42e‐24; *Grip1*, 5.96e‐18; *Sema6d*, 7.02e‐27; *Kirrel3*, 1.46e‐04. (D) Genome browser tracks of chromatin accessibility near *Ebb4*, *Grip1*, and *Robo1*. Genes in the sense and antisense directions are shown in red and blue, respectively. The tracks are shown for young and aged cells separately. (E) TF motif enrichment analysis on aged‐down DARs in OLIGO. The enriched TF motifs are distributed by their sorted rank on the *x‐*axis and −log_10_ (*q*‐value) on the *y‐*axis for significance. CREB5 is highlighted in red. (F) Bar plot of the enriched GO biological process terms for CREB5 motif enriched in aged‐downregulated DARs of OLIGO. The sequence logo was modified from JASPAR2024 web after querying MA0840.1 (https://jaspar.elixir.no). (G) Hypothetical model scheme for the potential function of CREB5 in the aging process of OLIGO. Downregulation of CREB5 possibly results in transcriptional repression of target genes involved in axon guidance (representative genes: *Dcc*, *Ank3*), differentiation (representative gene: *Dusp10*), cell communication (representative gene: *Ank3*), and learning (representative gene: *Cux2*). This transcriptomic reprogramming might contribute to aging‐associated functional decline in OLIGO and in neuron/OLIGO communication, thereby influencing neuronal function.

To elucidate the biological implications of the observed aging‐related changes, we conducted gene ontology (GO) analyses for aging‐associated DEGs and nearby genes of aging DARs, including promoters and enhancers (Methods). Several neuronal genes in glial cells, including OLIGO and ASTRO, were affected by aging at both levels of gene expression and chromatin accessibility (Figures 3A,B and S4A; Table S3). For example, we observed that DARs related to vesicle‐mediated transport in synapse and neurotransmitter secretion displayed decreased accessibility in ASTRO, showing a correlation with decreased expression of the corresponding genes (Figure 3B). Specifically, several synaptic plasticity‐related genes showed significant age‐associated changes in both expression and chromatin accessibility: In OLIGO, *Cdh8*, *Prkca*, and *Robo1* were upregulated, while *Kndc1, Ank3*, and *Creb5* were downregulated (Figure 3C,D). In ASTRO, *Brinp3*, *Fgf13*, and *Erbb4* were upregulated, whereas *Kirrel3*, *Sema6a*, and *Grip1* were downregulated with aging (Figure 3C,D).

To characterize the regulatory implications of these DARs, we performed a motif enrichment analysis and a GO analysis on TFs whose motifs were enriched in these DARs, using all human transcription factors as the background (Figures 3E, S4B–D, Table S3). Neuronal function‐associated terms were detected together with aging‐related terms, including immune response and apoptosis (Figure S4D). Specifically, regulation of neurogenesis and neuron differentiation was enriched among TF motifs enriched on aged–up DARs in OLIGO, while neuron differentiation and axon guidance were enriched in ASTRO. These findings suggest a significant reconfiguration of gene regulatory programs in glia, highlighting their potential role in contributing to the functional decline observed in aging neurons.

Among these TFs, we focused on CREB5 due to its significant downregulation in expression level in aged OLIGO (Figure 3C). Putative CREB5 target genes, defined by the presence of CREB5 motifs in aged–down DARs, were enriched for processes associated with learning, synaptic transmission, axon guidance, as well as oligodendrocyte differentiation, interleukin‐5 production, and alternative splicing regulation (Figure 3F). Notably, neuronal function‐related DEGs, including *Cux2*, *Ank3*, *Dcc*, and *Dusp10*, were among these putative targets (Figure 3C). While there is currently no direct experimental evidence of CREB5 binding to *Ank3* in OLIGO, a previous study showed that oligodendrocyte–specific deletion of *Ank3*, which encodes glial AnkG, impairs axoglial interactions during aging and affects mouse behaviors, including motor learning, sociability, and depression (Ding et al. 2024). These findings suggest a dysregulated perisynaptic function involved in neuron–glia communication in aging and support a pivotal role of glia, such as astrocytes, which have been shown to modulate the synaptic environment, thereby influencing neuronal function and plasticity during aging (Popov et al. 2021).

In addition to changes observed in OLIGO, we also identified aging‐associated regulatory alterations in ASTRO. Specifically, aging‐up DARs were enriched for NR6A1 motifs (Figure S4B), and *Nr6a1* expression was increased in aged ASTRO (Figure S4E). These results suggest a potential role of the NR6A1‐associated regulatory program in astrocyte aging.

**FIGURE 4:**
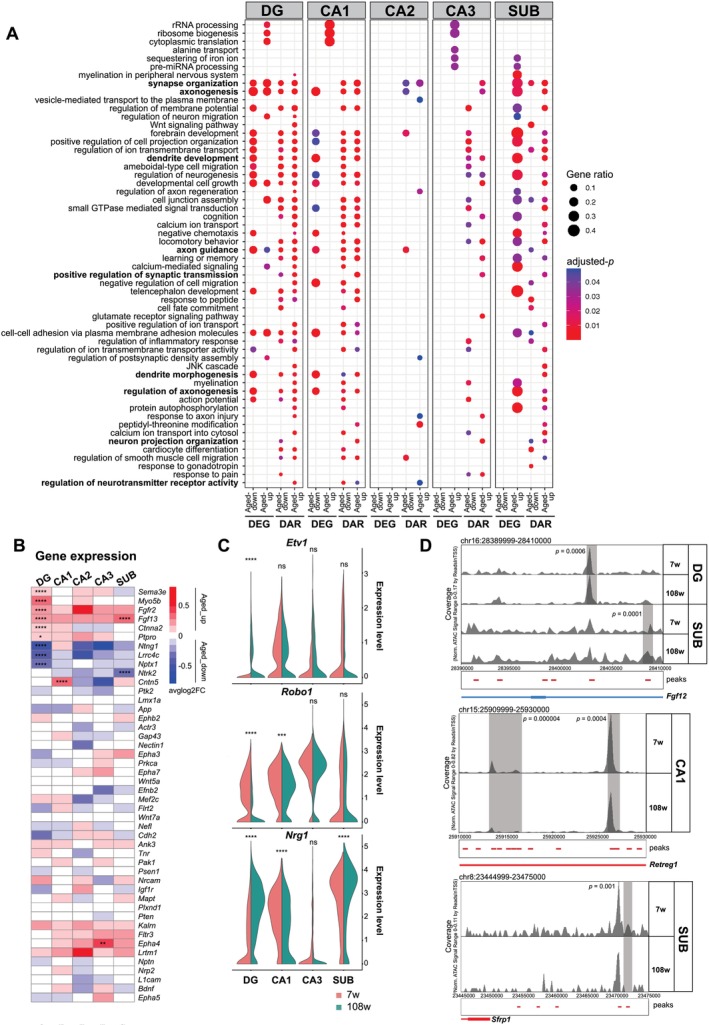
Chromatin accessibility–level dysregulations recapitulated aging features in neurons. (A) Gene ontology enrichment analysis for biological processes in neuronal subtypes based on significant DEGs and nearest genes of DARs. Gene ratio is indicated by the size of circles, and the color intensity indicates significance by adjusted *p*‐value. (B) Heatmap of averaged log‐fold change of expression genes representative for axonogenesis and synaptic organization‐related terms in (A), in young and aged cells of neuronal subtypes. Adjusted *p*‐values obtained with Seurat's *FindMarkers* function with the MAST algorithm using a hurdle model as a statistical test are shown (ns: Non‐significant; *Adjusted‐*p* < 0.05; **Adjusted‐*p* < 0.01; ***Adjusted‐*p* < 0.001; ****Adjusted‐*p* < 0.0001; Table S2). Adjusted *p*‐values calculated for genes showing a significant value are as follows: *Sema3e*, 2.59e‐17 in DG; *Myo5b*, 3.03e‐13 in DG; *Fgfr2*, 2.09e‐12 in DG; *Fgf13*, 2.41e‐10 in DG, 2.39e‐6 in SUB; *Ctnna2*, 4.25e‐05 in DG; *Ptpro*, 0.02 in DG; *Ntng1*, 5.33e‐74 in DG; *Lrrc4c*, 1.56e‐23 in DG; *Nptx1*, 2.46e‐20; *Ntrk2*, 6.04e‐06; *Cntn5*, 4.28e‐05. For the remaining genes, adjusted *p*‐value was equal to 1, or the gene was not detected in the resulting DEG list. (C) Violin plot of average gene expression for representative synaptic genes, *Robo1*, *Etv1*, and *Nrg1* in neuronal subtypes of young and aged mice. Adjusted *p*‐values obtained with Seurat's *FindMarkers* function with the MAST algorithm using a hurdle model as a statistical test are shown (ns: Non‐significant; *Adjusted‐*p* < 0.05; **Adjusted‐*p* < 0.01; ***Adjusted‐*p* < 0.001; ****Adjusted‐*p* < 0.0001; Table S2). Adjusted *p*‐values are as follows: *Etv1*, 1.98e‐13 in DG; *Robo1*, 0.001 in CA1, 1.21e‐161 in DG; *Nrg1*, 3.21e‐180 in DG, 1.82e‐25 in CA1, 1.23e‐05 in SUB. (D) Genome browser tracks of chromatin accessibility near *Fgf12*, *Retreg1*, and *Sfrp1*. Genes in the sense and antisense directions are shown in red and blue, respectively. The tracks are shown for young and aged cells separately. Significant peaks are highlighted in gray. *p*‐values obtained in edgeR's DAR analysis are shown (Table S2; Methods).

Furthermore, enhancers associated with DNA integrity and mRNA splicing‐related genes exhibited aging‐related changes in OLIGO, while enhancers associated with telomere organization and double‐strand break repair–related genes were affected in ASTRO (Figure 3A,B). These results suggest that global aging features, including mRNA splicing, telomere shortening, and altered DNA integrity, might be regulated at the enhancer level in glial cells, which were not detected in neuronal subtypes (Figure 4).

### 2.4 Chromatin Accessibility–Level Dysregulations Recapitulate Aging Features in Neurons

To investigate aging‐associated dysregulations in neurons, we analyzed changes in both chromatin accessibility and gene expression. GO term analysis unveiled common features shared by neurons and glial cells, notably in synapse organization and axonogenesis (Figures 3 and 4A; Table S4). Within neurons, DAR analysis detected consistent enrichment for terms related to synapse organization, axonogenesis, learning, and cognition across major cell types, which were not necessarily enriched in DEG analysis (Figures 4A,B and S5A). Notably, axon guidance and synaptic plasticity–genes such as *Robo1* and *Nrg1* were commonly altered with aging, while others, such as *Etv1*, showed cell type–specificity (e.g., DG; Figure 4C). *Robo1* was previously shown to exhibit reduced chromatin accessibility in its putative enhancers and promoters with hippocampal aging (Figure 2E; Zhang et al. 2022).

Distinct cell type–specific alterations also emerged from chromatin accessibility analysis (Figure 4A). Notably, SUB and DG neurons exhibited increased accessibility in gene loci associated with the JNK cascade (e.g., *Fgf12*), involved in axonal response and synapse growth, highlighting their distinctive susceptibility to aging (Figure 4D; Etter et al. 2005; Raivich et al. 2004). CA1 neurons displayed an altered transcriptome related to rRNA metabolism, dendrite development, and axon guidance. On the other hand, a putative enhancer and the promoter of *Retreg1*, known for its neuroprotective role (Mo et al. 2020), were less accessible in aged CA1 neurons, but the expression was not altered (Figures 4D and S5C). SUB neurons exhibited alterations in potential regulator loci located outside the gene body, at sites associated with Wnt signaling (e.g., *Sfrp1*), indicating their specific responses to aging (Figure 4D).

Given that DEG analysis did not capture many synaptic plasticity‐related terms, particularly in CA1, CA2, and CA3 neurons (Figure 4A,B; Table S4), which were detected at the chromatin level, these findings indicate that aging‐related transcriptomic changes only partially recapitulate aging features observed in vivo, including axonogenesis and dendrite morphogenesis. In contrast, chromatin accessibility profiling uncovered broader age‐related remodeling at regulatory regions linked to genes involved in synapse organization, synaptic transmission, learning or memory, and neurotransmitter receptor activity. Notably, many of these chromatin alterations occurred without accompanying gene expression changes, indicating the presence of a “susceptible” chromatin state in aging neurons (Figure 4B). While gene expression remains largely stable under basal conditions, the regulatory chromatin landscape is reshaped with age, potentially predisposing these neurons to stressors and to age‐related functional decline and diseases. This phenomenon aligns with previous findings in other systems, such as the observation of a primed chromatin state in immune genes within oligodendrocytes from control brains in multiple sclerosis (Meijer et al. 2022).

To further validate these findings, we examined aging DEGs in excitatory neurons using publicly available whole‐cell RNA‐seq datasets, which preserve more cytoplasmic transcripts compared to single‐nucleus approaches (Table S4; Methods; Ogrodnik et al. 2021). Consistent with our observations, these datasets lacked enrichment for synaptic function–related terms, but instead were dominated by signatures of inflammation and immune response (Figure S5B). Additionally, assessment of epigenetic marks revealed no significant changes in overall H3K27ac (an active chromatin mark) in NeuN–sorted neuronal nuclei from young or aged hippocampus, suggesting an unchanged profile of basal H3K27–acetylation in aging hippocampus (Figure S5D).

However, at the level of representative “susceptible” genes described above, the public single cell epigenetic analysis in aging hippocampus showed that the heterochromatin mark H3K9me3 was increased at aged‐down DARs (e.g., *L1cam*) in CA1, and reduced at aged‐up DARs (e.g., *Efnb2*), in line with the patterns of the observed chromatin accessibility changes (Figure S5E; Zhang et al. 2022). We also confirmed the increased tendency of deposition of H3K9me3 on *App*, which harbored an aged‐down DAR, and increased H3K27ac on *Psen1*, for which we identified an aging‐up DAR, by ChIP–qPCR using whole hippocampal nuclei (Figure S5F,G). These findings support the notion that specific chromatin modifications partially underlie the aging‐related regulatory landscape in hippocampal neurons.

Taken together, these results indicate that chromatin accessibility changes in neurons potentially facilitate aging‐related functional decline, before gene expression shifts become visible, particularly at genes involved in synaptic plasticity, axonogenesis, and learning. This susceptible chromatin state may predispose hippocampal neurons to age‐related brain dysfunctions and disease, especially under additional stress. Altogether, these findings highlight both shared and cell type–specific aging vulnerabilities and underscore the importance of chromatin‐level regulation in neuronal aging.

### 2.5 BACH2 Activity Reduced in Aged DG Neurons

**FIGURE 5:**
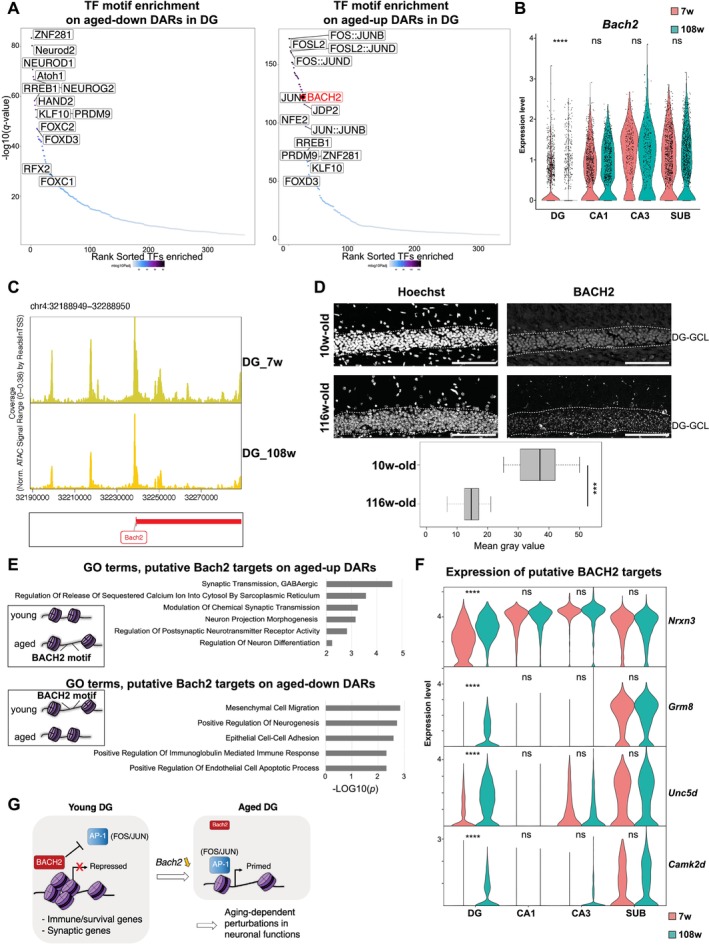
BACH2 activity reduced in aged DG neurons. (A) TF motif enrichment analysis on aged‐down DARs (left) and aged‐up DARs (right) in DG neurons. The enriched TF motifs are distributed by their sorted rank on the *x*‐axis and −log_10_ (*q*‐value) on the *y*‐axis for significance. (B) Violin plot of normalized gene expression for *Bach2* in each cell of neuronal subtypes split by their age. Adjusted *p*‐values obtained with Seurat's *FindMarkers* function with the MAST algorithm using a hurdle model as a statistical test is shown (ns, Non‐significant; ****Adjusted‐*p* < 0.0001; Table S2; 2.42e‐40 in DG). (C) Genome browser tracks visualization of *Bach2* loci in DG neurons. The tracks are shown for young and aged DG cells separately. (D) Immunostaining for anti‐BACH2 in young (10‐week‐old) and aged (116‐week‐old) hippocampus. Images were cropped to DG area. Nuclei were counterstained with Hoechst 33342. Each box represents the distribution of the mean gray value of the individual mice in each group (*n* = 2 mice for young; *n* = 3 mice for aged). Scale bars, 100 μm. ***p < 0.001 (*p =* 0.00025). DG‐GCL: Dentate gyrus–granular cell layer. (E) Gene ontology enrichment analysis for biological processes in DG neurons based on nearest genes of young and aged DARs with a BACH2 motif. The *x‐*axis indicates significance by −log_10_ (adjusted *p*‐value). (F) Violin plot of normalized gene expression for representative putative BACH2 targets, *Nrxn3*, *Grm8*, *Unc5d*, and *Camk2d*. Adjusted *p*‐values obtained with Seurat's *FindMarkers* function with the MAST algorithm using a hurdle model as a statistical test are shown (ns: Non‐significant; ****p < 0.0001; Table S2). Adjusted *p*‐values are as follows: *Nrxn3*, 3.65–182 in DG; *Grm8*, 4.51e‐136 in DG; *Unc5d*, 1.07e‐126 in DG; *Camk2d*, 1.78e‐135 in DG. (G) Hypothetical model scheme for the potential function of BACH2 in the aging process of DG neurons. Downregulation of BACH2 possibly allows the aberrant activation of AP‐1 target genes involved in immune response, survival, or synaptic function. This transcriptomic reprogramming might contribute to aging‐associated functional decline in neurons.

We then focused on DG neurons as they exhibited the most significant transcriptomic and chromatin level changes with aging among all neuron subtypes, including neuronal function‐related genes (Figure 4A). To uncover cis‐regulatory mechanisms underlying DG aging, we performed motif enrichment analysis on aging DARs, revealing a pronounced enrichment for motifs associated with neuron differentiation, axon development, axon guidance, and cellular response to calcium ions (Figure S6A,B; Table S5). Whereas aged‐up DARs in DG neurons were enriched in motifs of TFs consisting of the activator protein‐1 (AP‐1) complex, such as JUN family proteins, FOS, and FOSL2, and the BTB and CNC Homology (BACH) family, aged‐down DARs were enriched in NEUROD1/2 and NEROG2, which are involved in neurogenesis (Figure 5A). The AP‐1 complex, composed of cJUN, JUNB, JUND, cFOS, FOSB, FOSL1, FOSL2, BATF, and BATF3, can bind to MAF recognition elements to activate the expression of the target genes (Eferl and Wagner 2003; Shaulian and Karin 2002). By contrast, BACH1 and BACH2 transcription factors recognize a similar motif for repression in a competitive manner with the AP‐1 complex (Jang et al. 2017; Itoh‐Nakadai et al. 2014; Tsukumo et al. 2013). The AP‐1 complex plays a critical role in regulating gene expression in response to a variety of physiological and pathological stimuli, such as neuronal activation, and is involved in synaptic plasticity, memory formation, and response to stress (Alberini 2009; Sanyal et al. 2002). In these studies, while the function of the AP‐1 complex is well documented in neurons, the role of BACH2 is not well understood; its role in DNA damage, apoptosis, immune response, and proliferation has been documented in other contexts such as immune cells (Uittenboogaard et al. 2013; Liu and Liu 2022).

Notably, we observed a decreased expression of *Bach2* specifically in DG neurons, which exhibited the most notable changes in transcriptome among all TFs enriched on aged‐up DARs (Figures 5B and S6D). We hypothesized that the reduction of BACH2, a transcription factor involved in cellular stress responses, contributes to the decline in neuronal function and neuron death in DG neurons. The accessibility of the *Bach2* promoter, gene body, and putative enhancers also diminished in aged DG neurons (Figure 5C). We confirmed the decline of BACH2 expression in the aging hippocampus in vivo by immunohistochemistry (Figure 5D).

The GO analysis on BACH2 motif–containing aged‐up DARs in DG identified an enrichment of genes involved in synaptic transmission (e.g., *Nrxn3* and *Grm8*), apoptosis (e.g., *Unc5d*), and ion transporter activity (e.g., *Camk2d*; Figure 5E,F; Table S5). The expression of these putative target genes was significantly upregulated in DG neurons (Figures 5F and S6C). While the overall expression of the nearest genes of the BACH2 motif–containing aged‐up DARs averaged in each neuron subtype significantly increased in aged DG neurons, we observed the opposite tendency for the nearest genes of aged‐down DARs (Figure S6C). This suggests a dual and selective role for BACH2 in activating and repressing the downstream target genes, or a complementary effect of other factors, such as the AP‐1 complex.

To further assess the direct regulatory impact of BACH2 on its target loci, we performed ATAC‐seq on primary neurons overexpressing BACH2 via AAV delivery for 7 days (Figure S6E). We observed altered chromatin accessibility of several putative BACH2 target regions (54 regions, *p*‐value < 0.05; Table S5). Notably, the locus containing a BACH2 motif on *Camk2d*—an aged‐up DEG in DG—showed reduced accessibility upon BACH2 overexpression, supporting a chromatin condensing role for BACH2 at this site (Figure S6F).

Additionally, we also observed widespread changes in both the expression and motif activity of BACH2 across all human AD cell types (Figure S6G), underscoring the broader relevance of BACH2–mediated regulation in neurodegenerative diseases.

These findings suggest a role for BACH2 in the transcriptional landscape of DG neurons during aging. The diminished BACH2 expression and its motif activity correspond with upregulation of putative target genes involved in synaptic organization, apoptosis, and immune response, suggesting that BACH2 loss leads to derepression of its targets and contributes to aging‐associated functional decline in DG neurons (Figure 5G).

## 3 Discussion

In this study, we systematically characterized cell type–specific gene regulatory networks in the aging mouse hippocampus using single‐nucleus multiome sequencing. Our approach, for the first time in brain aging, integrated transcriptome and chromatin accessibility profiles from the same nuclei, enabling precise correlation of chromatin accessibility and transcriptional changes, and a clear distinction of neuronal subtypes, such as CA2 and CA3, which was not possible when using single modalities alone (RNA or ATAC). We revealed significant age‐dependent chromatin and transcriptome alterations across hippocampal cell types, highlighting differential susceptibility to aging, particularly in DG, CA1, SUB neurons, and glial cells.

We observed more extensive chromatin changes than transcriptome changes in excitatory neurons (DG, CA1–3, and SUB). Notably, chromatin accessibility changes at synaptic plasticity‐related loci often preceded transcriptomic alterations, supporting the existence of a susceptible chromatin state. While this state may not alter baseline transcription during normal aging, it could enable or amplify transcriptional responses when neurons are subjected to environmental changes, such as inflammation or oxidative damage. This susceptible state may, in part, result from the loss of heterochromatin domains (e.g., reduced H3K9me3 deposition), as previously observed in aged excitatory neurons (Zhang et al. 2022; Pérez et al. 2024) and observed in our study. Further investigation of the dynamics of chromatin remodeling and histone modification in different neuron subtypes at more continuous aging stages will be essential for clarifying how early epigenomic shifts drive neuronal aging.

Previous bulk transcriptomic and microarray studies reported global declines in neuronal and synaptic genes, with increased immune gene expression in aging brains, but lacked cell type resolution (Ham and Lee 2020; Loerch et al. 2008; Lu et al. 2004; Berchtold et al. 2008, 2013; Erraji‐Benchekroun et al. 2005; Bae et al. 2018; Naumova et al. 2012). Recent single‐cell studies, including ours, indicate that not all excitatory neuron subtypes exhibit alterations in neuronal function–related genes in their transcriptome with aging (Allen et al. 2023; Morabito et al. 2021). While the neuronal subtype distinction was not specifically addressed in these studies, our study has provided neuron subtype–specific alterations in gene expression and chromatin accessibility in the aging hippocampus; only DG and SUB neurons, along with glial cells, undergo significant synaptic gene alterations. Our findings thus suggest that changes in chromatin accessibility provide a more sensitive and cell type–specific reflection of synaptic plasticity–related dysregulation during hippocampal aging, particularly in cases where corresponding gene expression changes are not yet apparent. Furthermore, previous research on bulk tissue studies reported alterations of synaptic plasticity–related genes at the mRNA splicing level rather than the expression level (Stilling et al. 2014; Tollervey et al. 2011), underscoring the need to explore cell type–specific post‐transcriptional mechanisms and their relationship to chromatin state in normal cognitive aging and neurodegenerative diseases.

DG neurons were most affected at both chromatin and transcriptional levels, with dysregulation in genes related to synaptic plasticity and apoptotic pathways, which were possibly modulated by BACH2 and AP‐1. We observed that BACH2 overexpression in primary neurons reduced chromatin accessibility at the *Camk2d* locus, suggesting direct repression at the chromatin level (Figure S6E). We identified increased *Jun* and decreased *Bach2* expression, together with heightened motif activity for both factors in aged DG neurons (Figures 5A,B and S6D). Our findings align with reports of an enhanced activity of the JUN motif in aging and neurodegeneration (Zhang et al. 2022; Morabito et al. 2021). BACH2, known for its role in regulating immune responses and cellular stress, has been shown to decrease in expression with age in various tissues, including healthy human lymphocytes and lung, as well as the liver, kidney, and spleen of aged mice. Conversely, AP‐1 undergoes activation with inflammation, and its motif is enriched in aged neuronal lineages (Long et al. 2019; Karakaslar et al. 2023; Uittenboogaard et al. 2013; Zhang et al. 2022). Moreover, aging of pyramidal neurons is linked to increased excitability due to changes in membrane properties and synaptic function (Simkin et al. 2015; Vaillend et al. 2002), and aged memory–impaired animals display enhanced cFOS activity in response to neural stimulation (Haberman et al. 2017). While we attempted in vivo BACH2 knockout and ChIP assays, these approaches were technically challenging, underscoring the need for further methodological optimization to fully elucidate BACH2's role in neuronal aging. Declining BACH2 with age may allow activation of synaptic, apoptotic, and immediate early genes, implicating BACH2 as a regulator of neuronal excitability via modulation of AP‐1 targets. These findings highlight BACH2 as a promising therapeutic target for improving hippocampal function in aging and neurodegenerative diseases.

While this study focused on the male brain, emerging data suggest that aging trajectories can diverge by sex, with females showing greater microglial inflammatory responses (Achiro et al. 2024; Hadad et al. 2019; Li et al. 2023; Berchtold et al. 2008; Mangold et al. 2017). Additionally, recent studies report a sex‐specific shift in cell type proportions, including a decrease in excitatory neuron populations in aging male brains and an increase in microglia in females (Autio‐Kimura et al. 2024). Interestingly, similar to an aging‐dependent separation of OLIGO clusters we observed on UMAP, we also noted age‐ or disease‐related separation patterns in oligodendrocyte subtypes in transcriptomic datasets derived from female brains (Allen et al. 2023; Morabito et al. 2021). These findings suggest that while some age‐related regulatory features may be conserved across sexes, particularly in oligodendrocytes, other aspects, such as immune cell responses and neuronal vulnerability, may differ significantly. Future studies incorporating both sexes and leveraging cell type‐resolved epigenomic data will be essential to fully characterize the complexity of brain aging.

Finally, our dataset exhibited limitations in detecting immune‐related genes due to the profiling of nuclei instead of cells in most cell types, particularly in microglia, aligning with previous findings of the differential capacity of mRNA capture between nuclear and cellular compartments of microglia in single‐cell studies. Specifically, single‐nucleus transcriptome sequencing has been shown to fail to robustly detect microglial activation genes, leading to an underrepresentation of immune‐related transcripts when compared to single‐cell RNA sequencing (Thrupp et al. 2020). This underscores the need for technical considerations when interpreting immune‐related gene expression in nuclear versus cellular transcriptomic datasets.

## 4 Materials and Methods

### 4.1 Ethics Statement

All animals were housed and studied in compliance with protocols approved by the Animal Care and Use Committee of The University of Tokyo. The approval numbers are P25‐8 and P30‐4 from the Graduate School of Pharmaceutical Sciences, and 0421 and A2022IQB001‐06 from the Institute for Quantitative Biosciences. All procedures were followed in accordance with the University of Tokyo guidelines for the care and use of laboratory animals and ARRIVE guidelines.

### 4.2 Mouse Maintenance and Brain Tissue Samples

Wild‐type C57BL/6N male mice and pregnant female ICR mice were purchased from CLEA Japan. Some of the aged male mice were provided by the Foundation for Biomedical Research and Innovation at Kobe through the National BioResource Project of the Ministry of Education, Culture, Sports, Science and Technology (MEXT), Japan. All mice were maintained in a temperature‐ and humidity‐controlled environment (23°C ± 3°C and 50% ± 15%, respectively) under a 12‐h light/dark cycle. Animals were housed in sterile cages (Innocage, Innovive) containing bedding chips (PALSOFT, Oriental Yeast), at a density of two to six mice per cage, and provided irradiated food (CE‐2, CLEA Japan) and filtered water ad libitum. Male mice were group‐housed from the pup stage to minimize aggression throughout the study (National Research Council (US) 2011; Lidster et al. 2019). Animals used for sn‐multiome‐seq were euthanized at 7 and 108 weeks of age, with two males used at each age.

For validation with immunohistochemistry, mice were euthanized at 10 and 116 weeks. Mice were anesthetized with isoflurane and perfused with ice‐cold PBS followed by 4% paraformaldehyde (PFA) in PBS. Brains were postfixed in 4% PFA overnight at 4°C, rinsed in PBS, and incubated at 4°C in 10%, 20%, and 30% sucrose until they sank to the bottom of the tube before being frozen in optimal cutting temperature (O.C.T.) compound (Sakura) and stored at −80°C.

### 4.3 Library Preparation for Single Nuclei Multiome (RNA + ATAC) Sequencing

The protocol for nuclear isolation from brain tissues was adapted from a previous study (Frey et al. 2023; Bundo et al. 2016). All procedures were carried out on ice. Frozen hippocampi of young and aged mice were sequentially homogenized using a syringe with 23G and 27G needles in 500 μL of 54% Percoll in homogenizing buffer (50 mM Tris–HCl pH 7.4, 25 mM KCl, 5 mM MgCl_2_, and 250 mM sucrose). The homogenate was then mixed with 10% NP‐40 (final concentration, 0.1%), incubated on ice for 15 min, and mixed with 500 μL of homogenizing buffer. Then, a Percoll gradient was prepared in the following order: 100 μL of 35% Percoll in homogenizing buffer at the bottom layer, 200 μL of 31% Percoll in the middle, and 1 mL of homogenate (27% Percoll) on the top layer. The tube was centrifuged at 20,000 × *g* for 10 min at 4°C. After removing the debris from the top layer, nuclei were collected from the bottom layer and transferred to a new tube. The nuclear pellet was resuspended in PBS containing 0.02% BSA. The nuclei were counted and diluted to a concentration of 4000 nuclei per microliter in PBS with 0.02% BSA.

The nuclei were then used for library preparation, targeting the recovery of 4000 nuclei per sample using a Chromium system (10× Genomics, Next GEM Single Cell Multiome ATAC + Gene expression Reagent Bundle, PN‐1000283). The libraries were sequenced on the DNB‐seq platform to obtain 100‐bp‐end reads.

### 4.4 Alignment, Raw Processing, and Quality Control of Sc‐Multiome‐Seq Data

Fastq alignment to the reference genome (mm10), barcode processing, single‐cell counting, and read quantification and filtering were performed on each sn‐multiome‐seq library using the Cell Ranger‐Arc pipeline version 2.0 (10× Genomics). Intronic reads for all samples were included to quantify pre‐mRNA transcripts to account for unspliced nuclear transcripts. Cell Ranger‐Arc filtered count matrices were used for downstream analysis.

### 4.5 Processing, Cell Clustering, Visualization, and Cluster Annotation

ArchR version 1.0.2 was used for processing the paired snRNA‐seq gene expression data and snATAC‐seq fragment data for all samples (Granja et al. 2021). The ArchR framework was used for quality control filtering steps, single‐cell clustering, peak calling, and differentially accessible region analysis. ATAC fragments were read using the *createArrowFiles* function. Gene expression values were added using the *addGeneExpressionMatrix* function. Nuclei were excluded from downstream analysis if the TSS enrichment score was < 6, fewer than 3000 unique nuclear fragments, or missing matched RNA reads. Nuclei doublets were excluded with the *addDoubletScores* and *filterDoublets* functions of ArchR using TileMatrix of the snATAC‐seq. The removal of doublets resulted in filtering of 4% and 3.7% of the two respective replicates of 7‐week‐old samples, and that of 4% and 4.4% of the two respective replicates of 108‐week‐old samples. As a result, 15,480 nuclei were used for downstream analysis with a median TSS score of 13,441 and a median fragments per nucleus of 27,484.5.

Dimension reduction was performed using each GeneExpressionMatrix derived from snRNA‐seq and TileMatrix derived from snATAC‐seq using the *addIterativeLSI* function of ArchR with default parameters and resolution set to 0.2 for clustering. We used the *addCombinedDims* function to combine two‐modality (RNA and ATAC) dimension reductions into a single reduction. For each reduction, Uniform Manifold Approximation and Projection (UMAP) was performed with the default parameters of the *addUMAP* function. Cell clusters were generated using Seurat implemented in ArchR using the combined RNA and ATAC matrix with a resolution set to 0.8. Cell type annotation was performed based on gene scores and gene expression. Gene score represents gene expression and is calculated on the basis of chromatin accessibility at the gene body, promoter, and distal regulatory regions. We identified marker genes for each cluster using the *getMarkerFeatures* function using GeneScoreMatrix and GeneExpressionMatrix (Table S1). We further confirmed cluster identities by following known markers for each major cell type: *Snap25*, *Syt1*, and *Celf4* for general neuron markers; *Slc17a7* and *Satb2* for excitatory neurons; *Prox1* for DG neurons; *Mpped1*, *Wfs1*, and *Dcn* for CA1 neurons; *Sulf2* and *Nrip3* for CA3 neurons; *Dlx1* and *Gad1* for interneurons; *Sst*, *Vip*, *Lamp5*, and *Sncg* for interneuron subtypes; *Cldn11*, *Plp1*, and *Mbp* for oligodendrocytes; *Olig1*, *Pdgfra*, and *Sox10* for oligodendrocyte precursors; *Slc1a3*, *Slc1a2*, *Gjb6*, *Glul*, *Aqp4*, *Fgfr3*, *Aldh1l1*, and *Gfap* for astrocytes; *Cx3cr1* and *Itgam* for microglia; *Col1a2* and *Slc6a13* for vascular meningeal cells; *Flt1*, *Pecam1*, and *Cldn5* for endothelial cells; and *Ttr* for ependymal or choroid plexus cells. Next, we merged clusters displaying similar markers for DEG and DAR analyses.

Seurat version 4 was used for differential gene expression analysis on a total of 14,908 nuclei carried over from the ArchR object. The merged Seurat object containing ArchR‐filtered cells was log‐normalized and scaled with default parameters. Eighteen principal components and 0.8 of resolution were used for clustering and UMAP reduction using the *FindNeighbors* and *RunUMAP* functions of Seurat. We confirmed that the median of the percentage of mitochondrial transcripts in all carried‐over nuclei was 0.062, representing the healthy state of the samples.

### 4.6 Peak Calling and Annotation

Pseudo‐bulk replicates were generated for major cell types using the *addGroupCoverages* function of ArchR. The reproducible peak sets for each major cell type at different ages were named with default parameters using the *addReproduciblePeakSet* function implementing MACS2 (version 2.2.5) to create a fixed‐width peak size of 501 bp and iterative overlap peak merging on the basis of coverage data grouped by each major cell type, split by age. A total of 478,861 peaks were generated and used for downstream analysis. Peaks were required to be present in at least two pseudo‐bulk replicates. Using mm10 annotations from the BSgenome.Mmusculus.UCSC.mm10 package version 1.4.3 in R, we annotated the peak set of each cell type from young and aged samples into the following categories: promoter, exonic, intronic, and distal (Table S1). For the annotation of aging DAR peaks, we have used the *annotatePeak* function of the ChIPSeeker package in R (Table S2; Yu et al. 2015). For the annotation of promoter and enhancer regions in aging DAR peak lists, *bedtools intersect* was used (Quinlan and Hall 2010). For promoters, we have used the promoter list generated by the Cellranger‐Arc pipeline. For enhancers, we have withdrawn the file of enhancer‐gene interactions identified in the cortex from http://www.enhanceratlas.org/downloadv2.php.

### 4.7 Differentially Accessible Region Analysis and Finding Nearest Genes

EdgeR was employed to generate differentially accessible regions in each of the major cell types between young and aged cells (Chen et al. 2024). Cell type–focused analysis rather than subtype (cluster)‐focused one was chosen to increase the coverage of clusters. The LRT method was used to obtain the reliability of two replicates. DARs were considered significantly different if they had a *p*‐value < 0.001 in downstream analyses (Table S2).

Aging DARs were annotated using the *annotatePeaks* function of the ChIPSeeker package with default parameters (Yu et al. 2015). The column *geneId* of the output file was used to define the nearest genes (Table S2).

### 4.8 TF Motif Enrichment Analysis

TF motif enrichment analysis on aging DARs was performed using Simple Enrichment Analysis (SEA) version 5.5.2 (Bailey and Grant 2021). Bed files containing significant aging DARs obtained as described above were converted to fasta format using bedtools' getfasta, to use as input on SEA. Motif annotations were obtained using the JASPAR CORE (2022) vertebrates database with default parameters (Castro‐Mondragon et al. 2022). The TF motifs were considered significantly enriched if the *E*‐value (multiplication of *p*‐value by the number of motifs in the input) was < 10. The enriched TF motifs are ranked according to their *E*‐value. All motifs in the output files from SEA had a *q*‐value < 0.01 (Tables S3 and S4).

### 4.9 Annotation of Putative TF–Target Genes

FIMO version 5.5.0 was used as a motif search tool to find aging DARs containing the MA1101.2 meme motif for BACH2 and the MA0840.1 meme motif for CREB5 (Grant et al. 2011).

For BACH2 motif analysis, a total of 2383 sequences and 2021 sequences identified in the DAR analysis for DG neurons were applied for aged‐enriched and young‐enriched peaks, respectively. This analysis detected 1554 or 349 matched sequences among aged‐enriched or young‐enriched peaks, respectively (Table S5). We used ChIPseekeranno's annotatePeak function with default settings to annotate the genomic regions with the motif (Yu et al. 2015). This annotation identified 546 and 245 genes for aged‐enriched and young‐enriched peaks, respectively.

For CREB5 motif analysis, 2782 and 1756 sequences were used for aged‐enriched and young‐enriched peaks in OLIGO, respectively, and resultant matched sequences were annotated using ChIPseekeranno with default settings on the annotatePeak function (Table S3). The annotation of putative CREB‐targets identified 250 genes for aged‐enriched peaks and 197 genes for young‐enriched peaks.

### 4.10 Module Score Calculation for AD Genes and TF–Putative Target Genes

To calculate the average expression levels of AD and BACH2‐putative target genes at a single‐cell level, we applied the *AddModuleScore* function of ArchR, implementing the Seurat *addModuleScore* function on GeneExpressionMatrix for gene score or GeneExpressionMatrix for gene expression. We set the background number as 5 genes for signal normalization and bins to 25 for background calculation. This function generated a module score by calculating the average expression of each test gene group, subtracted by the aggregated expression of control gene sets. The Kruskal–Wallis test was performed for significance (ns: non‐significant, *p* > 0.05; *p ≤ 0.05; **p ≤ 0.01; ***p ≤ 0.001, ****p < 0.0001).

### 4.11 Differential Gene Expression Analysis

ArchR cluster assignments were added to Seurat metadata. The merged Seurat object was normalized to the sequencing depth and scaled with default parameters. DEG analysis was performed on ArchR clusters by the *FindMarkers* function in Seurat using the two‐hurdle model in DE testing implemented in the MAST algorithm to reduce false positives. A cell type–focused analysis rather than subtype (cluster)–focused one was chosen to increase the coverage of clusters. To regress out technical biases from different samples, the latent.vars = “batch” option was used to perform DE testing. Log_2_ (fold‐change) values of the average gene expression and adjusted *p*‐values were generated between young‐ and aged‐derived cells of each major cell type.

The list of DEGs for major cell types was generated, including all detected genes (Table S2). For downstream analyses, such as Gene Ontology analysis, the genes were filtered for adjusted *p* < 0.05.

To reveal the transcriptional distinction of NSC–like cluster 11 from other ASTRO clusters in Figure 1B, the DEG analysis between cluster 9 and 10 and cluster 11 using cells from young samples was performed by regressing out technical biases using the latent.vars = “batch” option. This DEG analysis resulted in 109 genes upregulated in cluster 11 (ASTRO2/NSC) and 129 genes upregulated in clusters 9 and 10 (Table S1; adjusted *p*‐value < 0.05).

### 4.12 Gene Ontology Term Enrichment Analysis

To identify the biological processes of each list of nearest genes of DARs, promoters, and enhancers on DARs, and DEGs in indicated cell types, clusterProfiler version 4.2.2 in R was used as an enrichment tool (Tables S3 and S4; Wu et al. 2021; Yu et al. 2012). Enrichplot version 1.14.2 in R was used to visualize functional enrichment results (Yu 2024). The top five terms with distinct gene sets are displayed (ranked by FDR).

For ASTRO subtype annotation, the resultant genes of DEG analysis were applied to the enrichR web (Chen et al. 2013). We queried GO Biological Process 2025 and displayed all detected terms (Table S1).

For DEGs identified in the Ogrodnik dataset, the list of significant DEGs (adjusted *p* < 0.05) was applied to the enrichR web (Table S4; Chen et al. 2013).

For TFs in glia, EnrichR web was used with the background defined as 1639 human TFs (Table S3; Lambert et al. 2018; Kuleshov et al. 2016). We queried GO Biological Process 2025 and displayed terms with a *p*‐value of less than 0.01. For TFs in DG, we queried biological processes in DAVID (Table S5; Huang et al. 2009). We noted that querying GO Biological Process 2025 on EnrichR web resulted in similar enriched terms as the terms detected in DAVID. Terms associated with the regulation of gene expression and transcription were not displayed in the figure (Tables S3 and S5).

For CREB5–putative target genes, clusterProfiler's enrichGO function was used to obtain associated biological processes (Yu et al. 2012; Table S3).

For BACH2–putative target genes, enrichR web was used (Chen et al. 2013). We queried GO Biological Process 2023 (Table S5).

### 4.13 Statistical Analysis

Fischer's exact test was used in Figure 2D. The Kruskal–Wallis test was performed for significance in violin plots in Figures S3B and S6C (ns: non‐significant; *p < 0.01; **p < 0.001; ***p < 0.0001). Significance in violin plots in Figures 3C, 4C, and 5F, and on the heatmap in Figure 4B was shown on the basis of adjusted *p*‐values obtained with Seurat's *FindMarkers* function with the MAST algorithm using a hurdle model as a statistical test (ns: non‐significant; *p < 0.05; **p < 0.01; ***p < 0.001; ****p < 0.0001). The pairwise Wilcoxon rank sum test was applied to find significant differences in BACH2 expression between young and aged samples. Statistical analysis of Western blot and ChIP‐qPCR results was performed in GraphPad Prism software, where statistical significance was determined at a threshold of *p* < 0.05 using an unpaired *t*‐test, and results are displayed as mean ± SD (*n* = 3 for WB; *n* = 4 for ChIP–qPCR).

## Author Contributions

**Merve Bilgic:** conceptualization, software, formal analysis, investigation, data curation, writing – original draft preparation, writing – review and editing, visualization, and project administration. **Rinka Obata, Vlada‐Iuliana Panfil, and Ziying Zhu:** formal analysis and investigation. **Mai Saeki:** formal analysis. **Yukiko Gotoh:** writing – review and editing, and supervision. **Yusuke Kishi:** conceptualization, writing – original draft preparation, writing – review and editing, supervision, project administration, and funding acquisition.

## Disclosure

Data and Code Availability: The sn‐multiome‐seq dataset was generated from raw sequencing data derived from two replicates of the hippocampus of 7‐week‐old and 108‐week‐old mice. Raw datasets (fastq files) or processed datasets have been deposited in the DNA Data Bank of Japan (DDBJ) Sequence Read Archive under the accession code PRJDB18626. The external Ogrodnik, Ortiz, and Zhang datasets are available in the GEO database (GSE161340 for Ogrodnik; GSE147747 for Ortiz; GSE187332 for Zhang; Ogrodnik et al. 2021; Ortiz et al. 2020; Zhang et al. 2022). BigWig files were used for the Zhang dataset without preprocessing. Additional methods and codes used in this study are available from the corresponding author upon reasonable request.

## Conflicts of Interest

The authors declare no conflicts of interest.

## Supporting information


**Data S1:** acel70233‐sup‐0001‐Supinfo.docx.

**Figure S1:** Single‐nucleus profiling of transcriptome and chromatin accessibility in the mouse hippocampus with aging. (A) Quality‐control metrics of each replicate in 7‐week‐old and 108‐week‐old samples after filtering low‐quality cells (Methods). From left to right, violin plots of log_10_(fragment numbers), gene numbers, and UMI numbers for each cell are shown. Rep: replicate. (B) The number of peaks and their annotation are shown for each replicate of cell types from young or aged samples. (C) Heatmap of top three markers showing gene expression and gene activity in each cluster by hierarchical ordering. Major cell types are indicated in columns above. (D) Violin plot of normalized gene expression for representative markers of astrocyte (*Aqp4*) or NSC markers (*Notch2*, *Cdk6*) in astrocyte clusters of Figure 1B. (E) Fraction of astrocyte clusters in each sample. *Y*‐axis represents the percentage of cells per sample (Table S1). (F) Gene ontology enrichment analysis for biological processes in astrocytes using C11–enriched genes, derived from the DEG analysis comparing cluster 11 and clusters 9 and 10. The *x*‐axis indicates significance by −log_10_(adjusted *p*‐value).
**Figure S2:** Quality control of clustering. (A) Visualization of cells on UMAP colored by RNA or ATAC clusters, with major cell types encircled according to their representative markers. (B) Heatmap of the confusion matrix representing the distribution of cells across clusters generated by RNA (rows) or ATAC (columns) modalities. Color scale represents the log_10_ (number of cells) for each RNA–ATAC cluster combination. (C) Boxplot of cluster purity for each cluster. The purity of neighborhood for each cell was computed, and cells were distributed according to their purity along the *y*‐axis; the median of each cluster is shown. (D) Visualization of cells colored by predicted cluster identities using Seurat's label transfer model with the Ortiz et al. dataset, and by cluster identities shown in Figure 1B. The heatmap shows the fraction of predicted identities in ArchR clusters and the color scale indicates the fractions of the range of 0–1.
**Figure S3:** Cell type–specific transcriptome and epigenome dynamics across hippocampal aging. (A) Percentage of genomic annotations of aging DARs in major cell types. The fractions were calculated separately for aged–upregulated DARs (aged‐up) and aged–downregulated DARs (aged‐down). (B) Module scores for each cell in young and aged DG, CA1–3, and SUB neurons. DEGs identified in CA1 and CA3 of the AD hippocampus in Miller et al. (2013) were used to calculate the module score, representing the average expression levels of these genes in each cell. The Kruskal–Wallis test was performed for significance (ns: non‐significant, *p* > 0.05; *p ≤ 0.05; **p ≤ 0.01; ***p ≤ 0.001, ****p < 0.0001).
**Figure S4:** Dysregulation of neuronal genes in glial cells during hippocampal aging. (A) Volcano plot for DEGs in oligodendrocytes, oligodendrocyte precursors, astrocytes, and microglial cells. The *x*‐axis indicates log_2_ (fold‐change) of average gene expression, and the *y*‐axis indicates significance in −log_10_ (adjusted *p*‐value). Vertical threshold is 0.25, and horizontal threshold is 3. (B, C) Transcription factor motif enrichment ranked by *E*‐values in (B) ASTRO, (C) OLIGO. (B) TF motif enrichment on aged‐up DARs and aged‐down DARs is shown separately on the left and right panels, respectively. (C) TF motif enrichment on aged‐up DARs is shown. The color intensity indicates significance by −log_10_ (adjusted *p*‐value). (D) Gene ontology enrichment analysis for biological processes of TFs enriched on aged‐up DARs in OLIGO or ASTRO. The *x*‐axis indicates significance by −log_10_ (*p*‐value). (E) Violin plot of normalized gene expression for *Nr6a1* in ASTRO split by age.
**Figure S5:** Chromatin accessibility–level dysregulations recapitulated aging features in neurons. (A) Volcano plot for DEGs in neuronal subtypes, including DG, CA1, CA3, and SUB. The *x*‐axis indicates log_2_ (fold‐change) of average gene expression, and the *y*‐axis indicates significance in −log_10_ (adjusted *p*‐value). Vertical threshold is 0.25, and horizontal threshold is 3. (B) Gene ontology enrichment analysis for biological processes using DEGs identified in Ogrodnik single‐cell datasets for the aging hippocampus. We identified 72 significant DEGs in excitatory neurons. The *x*‐axis indicates the significance by −log_10_ (*p*‐value). (C) Violin plot of gene expression for *Retreg1* in CA1 neurons split by age. (D) Western blot analysis of H3 and H3K27ac levels in hippocampal neurons from young and aged mice. Statistical significance was assessed using an unpaired *t*‐test (*p* = 0.26; *n* = 3 biological replicates from different brains). (E) Genome browser tracks displaying H3K9me3 signal at aging DAR loci for *L1cam* and *Efnb2* in CA1, visualized using IGV (version 2.15.4, https://igv.org/doc/desktop/). Separate tracks are shown for young (3 months) and aged (18 months) cells. BigWig files sourced from the Zhang dataset were loaded directly to IGV, allowing visualization without additional preprocessing of the original deposited dataset. The arrows on the left indicate the direction of enrichment of H3K9me3 at DAR‐loci; an age‐associated increase at *L1cam* and a decrease at *Efnb2*. The genomic region of aging DARs detected on these loci (aged‐down DAR on L1cam, aged‐up DAR on Efnb2) is shown on the corresponding genome browser tracks. (F) H3K9me3 ChIP–qPCR analysis of *App*, comparing young and aged hippocampal cells. Data are normalized to aged samples (*p* = 0.0351; *n* = 4 biological replicates from different brains). (G) H3K27ac ChIP–qPCR analysis of *Psen1*, comparing young and aged hippocampal cells. Data are normalized to aged samples (*p* = 0.0351; *n* = 4 biological replicates from different brains).
**Figure S6:** BACH2 activity reduced in aged DG neurons. (A, B) Gene ontology enrichment analysis for biological processes in neuronal subtypes of TF motifs enriched on aged‐down DARs (A) and aged‐up DARs (B). The *x*‐axis indicates the significance by –log_10_ (*p*‐value). (C) Violin plot of average expression scores for BACH2–putative target genes identified in Figure 5, in each cell of neuronal subtypes split by their age. (D) Violin plot of average gene expression for *Bach1* and AP‐1 components, *Jund*, *Jun*, *Fos*, and *Fosl2* in DG neurons split by their age of sample collection. (E) Representative Western blot analysis of BACH2 and GAPDH in primary neurons transduced with AAV vectors expressing BACH2‐GFP or control GFP. (F) Genome browser tracks displaying ATAC signals at Bach2 motif‐containing genomic loci for *Camk2d*, following BACH2 overexpression in primary hippocampal neurons. Separate tracks are shown for each replicate (three for both control and Bach2 overexpression). The location of the Bach2 motif, identified in Figure 5E (Table S5), is indicated. *N* = 3 biological replicates per group. (G) Violin plot of expression level (left) and motif enrichment (right) of Bach2 in human AD dataset. The plots were taken from https://swaruplab.bio.uci.edu/singlenucleiAD/ (Morabito et al. 2021). The Kruskal–Wallis test was performed for significance (ns: non‐significant, *p* > 0.05; *p ≤ 0.05; **p ≤ 0.01; ***p ≤ 0.001, ****p < 0.0001).

**Table S1:** Quality control, metadata, and cluster information, related to Figure 1.

**Table S2:** DEG and DAR analyses related to Figure 2.

**Table S3:** Gene Ontology analysis and TF motif enrichment related to Figure 3.

**Table S4:** Gene Ontology analysis related to Figure 4.

**Table S5:** TF and GO analyses related to Figure 5.

## Data Availability

The data that support the findings of this study are openly available in the DNA DataBank of Japan (DDBJ) at https://www.ddbj.nig.ac.jp/index‐e.html.
