# Supplementary material for: Age‐Associated Transcriptomic and Epigenetic Alterations in Mouse Hippocampus
Source: Aging Cell. 2025 Sep 28;24(11):e70233. doi: 10.1111/acel.70233 (PMC12608092; doi:10.1111/acel.70233)
Supplement: Supplementary file 1 — Data S1: acel70233‐sup‐0001‐Supinfo.docx. [file ACEL-24-e70233-s001.docx]

**SUPPORTING INFORMATION**

Results related to Figure 1:

**Characterization of ASTRO2/NSC Cluster**

Among ASTRO clusters, cluster 11 exhibited features characteristics of both immature astrocytes and neural stem cells (NSCs), such as higher expression of *Notch2* and *Cdk6*, compared to other ASTRO clusters, cluster 9 and 10 (Figure S1D–F; Table S1). Based on these properties, we designated this cluster as ASTRO2/NSC. Consistent with previous reports (Kuhn *et al*, 1996; Kalamakis *et al*, 2019), the proportion of ASTRO2/NSC cells are markedly reduced in aged hippocampus (comprising 0.7% in 7w replicate 1, 0.5% in 7w replicate 2, 0% in 108w replicate 1, 0.3% in 108w replicate 2; Figure S1E; Table S1).

**Enhanced Cell Type Resolution through Multimodal Integration of RNA and ATAC Data**

Notably, as reported in previous single-cell multiome studies on mouse skin, on human developing cortex, and on human peripheral blood mononuclear cells (Hao *et al*, 2021; Zhu *et al*, 2023; Ma *et al*, 2020), integrating RNA and ATAC data provided greater resolution in distinguishing cell types compared to using a single modality (Figure S2A–B). While single-cell RNA-seq captures gene expression profiles, it may not fully resolve closely related cell types or states, especially when transcriptional differences are subtle. Previous reports revealed that the regulatory elements of the genome, such as enhancers, are highly cell type-specific and can distinguish cell types (Buenrostro *et al*, 2015; Roadmap Epigenomics Consortium *et al*, 2015). Thus, a multimodal approach enhances the ability to distinguish between subtypes, as some cell states may exhibit similar RNA profiles but differ markedly in their epigenomic landscapes. For example, the CA2 cluster–characterized by a smaller number of cells in the mouse brain–could not be distinguished on UMAP using either RNA or ATAC modality alone (Figure S2A). However, integrative clustering revealed a clear separation between CA2 and CA3 neurons (Figure 1B). Additionally, only RNA data was sufficient to distinguish interneuron subtypes. ATAC data alone had ambiguous separation among excitatory neurons (Figure S2A): Some ATAC clusters (11, 14, and 17) co-expressed markers for DG, CA1, CA3, and SUB and did not correspond with a specific RNA cluster (Figure S2B). Overall, this integrative approach was especially pivotal in delineating neuronal subtypes, as joint profiling of chromatin accessibility and gene expression provided unique insights into excitatory neuron diversity (Figure 1B).

**Validation of Clustering Quality and Cell Type Annotation**

To ensure the accuracy of our clustering and cell type annotation, we performed additional validation analyses. First, we assessing whether over-clustering occurred by evaluating cluster separation based on the purity of the neighborhood for each cell as reflected; high–weighted purity proportions (median > 0.9) indicated the absence of over-clustering (Methods; Figure S2C). Next, we utilized Seurat’s label transfer method, which leverages prediction scores using a spatial transcriptomics reference dataset of the hippocampus, to validate our cluster annotation for hippocampal neurons (Figure S2D; Table S1; Ortiz et al., 2020; Stuart et al., 2019). The strong prediction scores supported the reliability of our identified neuronal subtypes, further reaffirming the utility of integrating RNA and ATAC profiles for comprehensive cell type characterization, especially for neuron subtypes.

**SUPPLEMENTARY DATA**

Supplementary Table 1. Quality control, metadata, and cluster information, related to Figure 1.

Supplementary Table 2. DEG and DAR analyses related to Figure 2.

Supplementary Table 3. Gene Ontology analysis and TF motif enrichment related to Figure 3.

Supplementary Table 4. Gene Ontology analysis related to Figure 4.

Supplementary Table 5. TF and GO analyses related to Figure 5.

**SUPPLEMENTARY MATERIALS AND METHODS**

**Overlap of DEGs and nearest genes of DARs**

Statistical significance of the overlap of genes between DEGs and nearest genes of DARs was computed by Fisher’s exact test using the GeneOverlap package in R (v1.38; Shen et al., 2024). The background for the gene overlap analysis was defined as the total number of genes detected in the merged Seurat object.

**Cluster purity analysis**

Bluster package in R was used to assess the cluster separation (Lun, 2023). Cluster purity of ArchR clusters was computed with the *neighborPurity* function in the UMAP expression space. Median purity greater than 0.9 indicated that cells from the same cluster are mainly surrounded by the other cells in the same cluster, reflecting that clusters are well-separated.

**Processing of external single-cell RNA-seq data sources**

To validate our DEG analysis on single-nuclei RNA-seq, we analyzed previously published single-cell RNA-seq studies of the aging brain. For the Ogrodnik dataset (Ogrodnik *et al*, 2021), two replicates of young and aged single-cell RNA-seq samples were withdrawn from the accession number GSE161340 of the NCBI Geo repository and merged in Seurat. Cells with more than 200 and less than 5,000 genes were removed. Twenty-five principal components were used for clustering and UMAP dimension reduction in the *FindNeighbors* and *RunUMAP* functions. The resolution was set to 0.8 in the *FindClusters* function, generating 23 clusters consisting of microglia, oligodendrocytes, endothelial cells, microglia, astrocytes, oligodendrocyte precursors, dentate gyrus neurons (DG), CA1 neurons, immature neurons, pericytes, choroid plexus cells, vascular cells, inhibitory neurons, and unknown cells. DG and CA1 clusters were further subsets for DEG analysis. The *FindMarkers* function was applied between young and aged cells of the major cell types for DE testing with default parameters. Significant DEGs with adjusted *p* < 0.05 were used for downstream analyses (72 genes).

For the Ortiz dataset (Ortiz *et al*, 2020), raw expression data and original metadata were withdrawn from accession number GSE147747 of the NCBI Geo repository. Hippocampal cells were subset using CA1slm, CA1so, CA1sp, CA1sr, CA2slm, CA2so, CA2sp, CA2sr, CA3slm, CA3slu, CA3so, CA3sp, CA3sr, DG-mo, DG-po, DG-sg, SUBd-m, SUBd-sp, SUBd-se, SUBv-sr, SUBv-m, SUBv-sp, SUBv-sr, PAR1, PAR2, PAR3, POST1, POST2, POST3, PRE1, PRE2, and PRE3 clusters. We performed sctransform-based normalization. We then performed the transfer labels method of Seurat by applying the *FindTransferAnchors* and *TransferData* functions using our dataset subset with neuronal clusters with sctransform-based normalization, which identified 587 anchors.

**Immunohistochemistry and confocal imaging**

Serial 14 µm coronal cryosections were cut on a Cryostat (TissureTek) and stored at −80°C until use. The sections were washed in 0.1 Tween-20 in TBS, and blocked in 0.1 Triton X-100 in TBS. The sections were stained with anti-Bach2 (rat; Abcam, ab243148, 1:250 dilution), anti–NeuN-488 (mouse; Millipore, MAB377X, 1:500 dilution) overnight at 4°C, followed by staining with donkey anti–rabbit Alexa Fluor 647 (Invitrogen, A32795, 1:1,000 dilution) and Hoechst (Invitrogen, H3570, 1:10,000 dilution), for 1 hour at room temperature. Primary antibodies were used in blocking buffer, and secondary antibodies and Hoechst were diluted in TBS with Tween-20. Slides were mounted with the mounting medium before imaging (Vectashield Vibrance Antifade Mounting Medium, Vector Laboratories, H-1700). Images were collected using an FV3000 confocal microscope (Olympus) and further processing was performed using FiJi (Schindelin *et al*, 2012). Confocal image stacks were stitched as two-dimensional maximum intensity projections using FiJi. The mean gray value was measured for the region of interest in the granular cell layer (GCL) of DG and subtract with the image background on each of 2 or 3 slices per brain. Statistical analysis was performed using pairwise Wilcoxon rank sum test.

**Western Blot**

Nuclear isolation from hippocampal tissue of young (7–weeks or 10–weeks old) and aged (108–weeks or 119–weeks old) mice was performed as described above. NeuN-positive nuclei were sorted using fluorescence-activated nuclei sorting (FANS, BD FACSMelody™). 50,000 nuclei for each sample were resuspended in 50 µl of 1x sample buffer (Nacalai Tesque, 09499–14), and heated at 95°C for 30 minutes. For primary neurons, cells were resuspended in 500 µl of 1x sample buffer. The hippocampal samples were further diluted 4 times in 1x sample buffer before loading 10µl of sample into Tris-Glycine (TGX) protein gel (BIORAD, 4561096) and subsequently transferred to a polyvinylidene fluoride (PVDF) membrane (0.2µm, BIORAD, 1704156). Membranes were washed in PBS before 30 minutes incubation in blocking buffer (Toyobo, NYPBR01). Membranes were incubated overnight at 4°C with primary antibodies; anti-Histone H3 (mouse monoclonal, Active Motif, 39763; 1:1000), anti-Histone H3 acetyl K27 (rabbit polyclonal, Abcam, ab4729; 1:500), anti-BACH2 (1:500; gift from Igarashi lab), anti-GAPDH (rabbit monoclonal CST, 2118S; 1:1000). Following washes with PBS with 0.1% Tween-20 (PBST), membranes were incubated with HRP-conjugated secondary antibodies for 30 minutes at room temperature (1:10,000). Protein expression was detected using chemiluminescent reagents (Immobilon Western Chemiluminescent HRP Substrate, Millipore, WBKLS0500) and quantified by ImageJ software, with results normalized to H3 for hippocampal samples. Three biological replicates each for young or aged mice were used. Results are displayed as mean ± SD.

**Primary neuron culture**

Primary cortical or hippocampal neurons were prepared from E16 ICR mouse embryos as described previously, with modifications (Hirabayashi *et al*, 2017). Dissected tissues were dissociated with neuron dissociation solutions (FujiFilm, 291-78001); first by incubating in enzyme solution at 37°C for 20 min, finally followed by gentle dissociation in Neurobasal medium (Gibco) supplemented with 2% B-27 (Thermo Fisher), 1% GlutaMAX (Gibco), and 2.5% FBS (Gibco). Cells were plated on poly-D-lysine–coated dishes (Sigma, 1 mg/mL) at a density of 0.5–1 × 10⁶ cells per 6-well dish. Cultures were maintained at 37°C in 5% CO₂. On day in vitro (DIV) 5 or 6, AAVs were added at 2,000 MOI per cell and cells were harvested 7 days post-transduction.

**Plasmid construction and AAV generation**

To generate the AAV overexpression construct (pAAV-hSyn-Bach2_P2A_EGFP, Addgene, 242200), the pAAV-hSyn-EGFP plasmid (Addgene, 50465, gifted by Dr. Takeuchi lab) was digested with NcoI to linearize the vector. The coding sequence of the Bach2 gene (ORF) and a P2A sequence were PCR-amplified with appropriate overhangs and inserted between the human synapsin (hSyn) promoter and EGFP by In-Fusion cloning (Takara Bio) according to the manufacturer’s instructions.

AAV production was done as previously described (Nakashima *et al*, 2022). Briefly, 293AAV (Cell Biolabs) cells were co-transfected with the AAV expression plasmid, AAV-DJ packaging plasmid (Addgene, 130878), and adenovirus helper plasmid (Addgene, 112867) using PEI-MAX (Cosmo Bio). After 72 hours, cells were harvested, and AAV particles were purified using the AAVpro Purification Kit Maxi (Takara, 6666) according to the manufacturer’s instructions. Viral titers were determined by quantitative PCR using SYBR Green and ITR-targeting primers. Aliquots were stored at -80°C until use.

**ATAC-seq analysis**

Primary hippocampal neurons were infected with AAV expressing BACH2 or control vector at, following the previously described protocol (Kujirai *et al*, 2025). The plated cells were washed with PBS, scraped into PBS, and centrifuged at 1,000 × g for 5 minutes at 4 °C. After the supernatant was removed, the cells were resuspended in 50 μl of lysis buffer (10 mM Tris-HCl, pH 7.4, 10 mM NaCl, 3 mM MgCl2, 0.1% NP-40) and centrifuged again at 1,000 × *g* for 5 minutes at 4 °C. The supernatant was discarded, and the pellet was resuspended in an ATAC reaction buffer containing 1 μl Tn5 transposase (Diagenode, C01070012), 10 μl Tagmentation Buffer (Diagenode, C01019043), and 9 μl water. The reaction was incubated at 37 °C for 30 minutes, followed by the addition of 100 μl GP1 buffer from the FastGene Gel/PCR Extraction Kit (Nippon Genetics, FG-91202). DNA was purified according to the manufacturer's protocol and amplified by PCR using Q5 Hot Start High-Fidelity 2× Master Mix (NEB, M0494) under the following conditions: 5 minutes at 72 °C, 30 seconds at 98 °C, followed by 12 cycles of 10 seconds at 98 °C and 20 seconds at 63 °C, with a final extension at 72 °C for 1 minute. The amplified DNA was purified using SPRI beads (Beckman Coulter, B23318).

The libraries were subjected to deep sequencing on the NextSeq2000 platform (Illumina) to produce 36-base paired-end reads. Approximately 20 million reads were obtained per sample. Adaptor sequences were trimmed using Cutadapt software, and the reads were subsequently mapped to the mouse genome (mm10) using Bowtie2 (Langmead *et al*, 2009). Uniquely mapped reads located in blacklisted regions, as determined by the Encyclopedia of DNA Elements (ENCODE) project (Dunham *et al*, 2012; Amemiya *et al*, 2019), as well as those on sex chromosomes, were removed using Bedtools (Quinlan & Hall, 2010). Duplicate reads were filtered out using the rmdup function in Samtools (Li *et al*, 2009). Chromatin accessibility signals within ±100 bp of BACH2 motifs were quantified using BAMscale software (Pongor *et al*, 2020), and statistical analysis were performed by edgeR software (Robinson *et al*, 2010). Chromatin accessibility at the *Camk2d* locus was visualized using the Integrated Genome Viewer (Robinson *et al*, 2011), focusing on the region encompassing the BACH2 motif and the corresponding differentially accessible region (DAR) identified in aging.

**ChIP-qPCR analysis**

Chromatin immunoprecipitation (ChIP) for H3K9me3 and H3K27ac was performed with slight modifications to the protocol described previously (Eto *et al*, 2020). Nuclei, prepared as mentioned earlier, were fixed using 1% formaldehyde and resuspended in RIPA buffer for sonication. The RIPA buffer composition was 10 mM Tris-HCl (pH 8.0), 1 mM EDTA, 140 mM NaCl, 1% Triton X-100, 0.1% SDS, and 0.1% sodium deoxycholate. Sonication was carried out using a Picoruptor (Diagenode) for 15 cycles of 30 seconds ON and 30 seconds OFF. The resulting lysates were diluted with RIPA buffer (50 mM Tris-HCl, pH 8.0, 150 mM NaCl, 2 mM EDTA, 1% NP-40, 0.1% SDS, and 0.5% sodium deoxycholate) for immunoprecipitation. To reduce non-specific binding, the lysates were incubated at 4 °C for 1 hour with Protein A/G Magnetic Beads (Pierce, #88803). Subsequently, the lysates were incubated overnight at 4 °C with Protein A/G Magnetic Beads preloaded with antibodies specific to H3K9me3 (MABI, 301-34833, 2 μg per reaction) or H3K27ac (Cell Signaling, 8173S, 2 μg per reaction). The beads were collected and washed three times using wash buffer (20 mM Tris-HCl, pH 8.0, 2 mM EDTA, 150 mM NaCl, 1% Triton X-100, and 0.1% SDS) and once with wash buffer containing 500 mM NaCl. The immune complexes were eluted from the beads by incubating them at 65 °C for 15 minutes in a solution comprising 10 mM Tris-HCl (pH 8.0), 5 mM EDTA, 300 mM NaCl, and 0.5% SDS. The eluted material underwent digestion with proteinase K (Nacalai, 29442-14) at 37 °C for over 6 hours, followed by cross-link reversal at 65 °C for an additional 6 hours. DNA was then extracted using phenol–chloroform–isoamyl alcohol and ethanol precipitation. After washing with 70% ethanol, the DNA was dissolved in water and analyzed using real-time PCR on a LightCycler 480 system (Roche) with QuantiNova reagents (Qiagen, 208054). The primer sequences were as follows: *App*: CGTTAAGGTTCCAGGGAAG and TCTCCAAATAGAGAGAACGAACT; *Psen1*: AACTGTTCAAATAAATACTGTGGAG and TTTGGTTGAGACTGCGATTC.

**SUPPLEMENTARY REFERENCES**

Amemiya HM, Kundaje A & Boyle AP (2019) The ENCODE Blacklist: Identification of Problematic Regions of the Genome. *Sci Rep* 9: 1–5

Buenrostro JD, Wu B, Litzenburger UM, Ruff D, Gonzales ML, Snyder MP, Chang HY & Greenleaf WJ (2015) Single-cell chromatin accessibility reveals principles of regulatory variation. *Nature* 523: 486–490

Dunham I, Kundaje A, Aldred SF, Collins PJ, Davis CA, Doyle F, Epstein CB, Frietze S, Harrow J, Kaul R, *et al* (2012) An integrated encyclopedia of DNA elements in the human genome. *Nat 2012 4897414* 489: 57–74

Eto H, Kishi Y, Yakushiji-Kaminatsui N, Sugishita H, Utsunomiya S, Koseki H & Gotoh Y (2020) The Polycomb group protein Ring1 regulates dorsoventral patterning of the mouse telencephalon. *Nat Commun 2020 111* 11: 1–17

Hao Y, Hao S, Andersen-Nissen E, Mauck III WM, Zheng S, Butler A, Lee MJ, Wilk AJ, Darby C, Zager M, *et al* (2021) Integrated analysis of multimodal single-cell data Graphical abstract. *Cell* 184: 3573-3587.e29

Hirabayashi Y, Kwon SK, Paek H, Pernice WM, Paul MA, Lee J, Erfani P, Raczkowski A, Petrey DS, Pon LA, *et al* (2017) ER-mitochondria tethering by PDZD8 regulates Ca2+ dynamics in mammalian neurons. *Science (80- )* 358: 623–630

Kalamakis G, Brüne D, Ravichandran S, Bolz J, Fan W, Ziebell F, Stiehl T, Catalá-Martinez F, Kupke J, Zhao S, *et al* (2019) Quiescence Modulates Stem Cell Maintenance and Regenerative Capacity in the Aging Brain. *Cell* 176: 1407-1419.e14

Kuhn HG, Dickinson-Anson H & Gage FH (1996) Neurogenesis in the dentate gyrus of the adult rat: age-related decrease of neuronal progenitor proliferation. *J Neurosci* 16: 2027

Kujirai T, Echigoya K, Kishi Y, Saeki M, Ito T, Kato J, Negishi L, Kimura H, Masumoto H, Takizawa Y, *et al* (2025) Structural insights into how DEK nucleosome binding facilitates H3K27 trimethylation in chromatin. *Nat Struct Mol Biol 2025*: 1–10

Langmead B, Trapnell C, Pop M & Salzberg SL (2009) Ultrafast and memory-efficient alignment of short DNA sequences to the human genome. *Genome Biol* 10: 1–10

Li H, Handsaker B, Wysoker A, Fennell T, Ruan J, Homer N, Marth G, Abecasis G & Durbin R (2009) The Sequence Alignment/Map format and SAMtools. *Bioinformatics* 25: 2078

Lun, A. 2023. “bluster: Clustering Algorithms for Bioconductor.” R package version 1.12.0, [Bioconductor - bluster](https://bioconductor.org/packages/release/bioc/html/bluster.html).

Ma S, Zhang B, LaFave LM, Earl AS, Chiang Z, Hu Y, Ding J, Brack A, Kartha VK, Tay T, *et al* (2020) Chromatin Potential Identified by Shared Single-Cell Profiling of RNA and Chromatin. *Cell* 183: 1103-1116.e20

Morabito S, Miyoshi E, Michael N, Shahin S, Martini AC, Head E, Silva J, Leavy K, Perez-Rosendahl M & Swarup V (2021) Single-nucleus chromatin accessibility and transcriptomic characterization of Alzheimer’s disease. *Nat Genet* 53: 1143–1155

Nakashima M, Ikegaya Y & Morikawa S (2022) Genetic labeling of axo-axonic cells in the basolateral amygdala. *Neurosci Res* 178: 33–40

Ogrodnik M, Evans SA, Fielder E, Victorelli S, Kruger P, Salmonowicz H, Weigand BM, Patel AD, Pirtskhalava T, Inman CL, *et al* (2021) Whole-body senescent cell clearance alleviates age-related brain inflammation and cognitive impairment in mice. *Aging Cell* 20: 1–16

Ortiz C, Navarro JF, Jurek A, Märtin A, Lundeberg J & Meletis K (2020) Molecular atlas of the adult mouse brain. *Sci Adv* 6

Pongor LS, Gross JM, Vera Alvarez R, Murai J, Jang SM, Zhang H, Redon C, Fu H, Huang SY, Thakur B, *et al* (2020) BAMscale: Quantification of next-generation sequencing peaks and generation of scaled coverage tracks. *Epigenetics and Chromatin* 13: 1–13

Quinlan AR & Hall IM (2010) BEDTools: a flexible suite of utilities for comparing genomic features. *Bioinformatics* 26: 841–842

Roadmap Epigenomics Consortium, Kundaje A, Meuleman W, Ernst J, Bilenky M, Yen A, Heravi-Moussavi A, Kheradpour P, Zhang Z, Wang J, *et al* (2015) Integrative analysis of 111 reference human epigenomes. *Nat 2015 5187539* 518: 317–330

Robinson JT, Thorvaldsdóttir H, Winckler W, Guttman M, Lander ES, Getz G & Mesirov JP (2011) Integrative genomics viewer. *Nat Biotechnol 2011 291* 29: 24–26

Robinson MD, McCarthy DJ & Smyth GK (2010) edgeR: a Bioconductor package for differential expression analysis of digital gene expression data. *Bioinformatics* 26: 139–140

Schindelin J, Arganda-Carreras I, Frise E, Kaynig V, Longair M, Pietzsch T, Preibisch S, Rueden C, Saalfeld S, Schmid B, *et al* (2012) Fiji: an open-source platform for biological-image analysis. *Nat Methods 2012 97* 9: 676–682

Shen, L., and Sinai ISoMaM. 2024. “GeneOverlap: Test and Visualize Gene Overlaps.” R Package Version 1.40.0.

Stuart T, Butler A, Hoffman P, Hafemeister C, Papalexi E, Mauck WM, Hao Y, Stoeckius M, Smibert P & Satija R (2019) Comprehensive Integration of Single-Cell Data. *Cell* 177: 1888-1902.e21

Zhu K, Bendl J, Rahman S, Vicari JM, Coleman C, Clarence T, Latouche O, Tsankova NM, Li A, Brennand KJ, *et al* (2023) Multi-omic profiling of the developing human cerebral cortex at the single-cell level. *Sci Adv* 9

**SUPPLEMENTARY FIGURE LEGENDS**

Figure S1. **Single-nuclei profiling of transcriptome and chromatin accessibility in the mouse hippocampus with aging.**

**A**. Quality-control metrics of each replicate in 7-week-old and 108-week-old samples after filtering low-quality cells (Methods). From left to right, violin plots of log_10_(fragments numbers), gene numbers, and UMI numbers for each cell are shown. Rep: replicate.

**B**. Number of peaks and their annotation is shown in each replicate of cell types from young or aged samples.

**C**. Heatmap of top three markers showing gene expression and gene activity in each cluster by hierarchical ordering. Major cell types are indicated in columns above.

**D**. Violin plot of normalized gene expression for representative markers of astrocyte (*Aqp4*) or NSC markers (*Notch2*, *Cdk6*) in astrocyte clusters of Figure 1B.

**E**. Fraction of astrocyte clusters in each sample. *Y*−axis represents the percentage of cells per sample (Table S1).

**F**. Gene ontology enrichment analysis for biological processes in astrocytes using C11−enriched genes, derived from the DEG analysis comparing cluster 11 and clusters 9 and 10. The *x*–axis indicates significance by −log_10_(adjusted *p*-value).

Figure S2. **Quality control of clustering.**

**A**. Visualization of cells on UMAP colored by RNA or ATAC clusters, with major cell types encircled according to their representative markers.

**B**. Heatmap of the confusion matrix representing the distribution of cells across clusters generated by RNA (rows) or ATAC (columns) modalities. Color scale represents the log_10_(number of cells) for each RNA–ATAC cluster combination.

**C**. Boxplot of cluster purity for each cluster. The purity of neighborhood for each cell was computed and cells are distributed according to their purity along the *y*–axis; the median of each cluster is shown.

**D**. Visualization of cells colored by predicted cluster identities using Seurat’s label transfer model with the Ortiz et al. dataset, and by cluster identities shown in Figure 1B. The heatmap shows the fraction of predicted identities in ArchR clusters and the color scale indicates the fractions of the range of 0 to 1.

Figure S3. **Cell type–specific transcriptome and epigenome dynamics across hippocampal aging.**

**A**. Percentage of genomic annotations of aging DARs in major cell types. The fractions were calculated separately in aged–upregulated DARs (aged-up) and aged–downregulated DARs (aged-down).

**B**. Module scores for each cell in young and aged DG, CA1–3, and SUB neurons. DEGs identified in CA1 and CA3 of AD hippocampus in Miller et al. were used to calculate module score, representing the average expression levels of these genes in each cell. The Kruskal–Wallis test was performed for significance (ns: non-significant, *p* > 0.05; *: *p* <= 0.05; **: *p* <= 0.01; ***: *p* <= 0.001, ****: *p* < 0.0001).

Figure S4. **Dysregulation of neuronal genes in glial cells during hippocampal aging.**

**A**. Volcano plot for DEGs in oligodendrocytes, oligodendrocyte precursors, astrocytes, and microglial cells. The *x*−axis indicates log_2_(fold-change) of average gene expression, and the *y*−axis indicates significance in −log_10_(adjusted *p*-value). Vertical threshold is 0.25 and horizontal threshold is 3.

**B-C**. Transcription factor motif enrichment ranked by *E*-values in B. ASTRO, C. OLIGO. B. TF motif enrichment on aged-up DARs and aged-down DARs are shown separately on left and right panels, respectively. C. TF motif enrichment on aged-up DARs are shown. The color intensity indicates significance by −log_10_(adjusted *p*-value).

**D**. Gene ontology enrichment analysis for biological processes of TFs enriched on aged-up DARs in OLIGO or ASTRO. The *x*−axis indicates significance by −log_10_(*p*-value).

**E**. Violin plot of normalized gene expression for *Nr6a1* in ASTRO split by age.

Figure S5. **Chromatin accessibility–level dysregulations recapitulated aging features in neurons.**

**A**. Volcano plot for DEGs in neuronal subtypes including DG, CA1, CA3, and SUB. The *x*-axis indicates log_2_(fold-change) of average gene expression, and the *y*-axis indicates significance in −log_10_(adjusted *p*-value). Vertical threshold is 0.25 and horizontal threshold is 3.

**B**. Gene ontology enrichment analysis for biological processes using DEGs identified in Ogrodnik single-cell datasets for the aging hippocampus. We identified 72 significant DEGs in excitatory neurons. The *x*-axis indicates the significance by −log_10_(*p*-value).

**C**. Violin plot of gene expression for *Retreg1* in CA1 neurons split by age.

**D**. Western blot analysis of H3 and H3K27ac levels in hippocampal neurons from young and aged mice. Statistical significance was assessed using an unpaired t-test (*p* = 0.26; n = 3 biological replicates from different brains).

**E**. Genome browser tracks displaying H3K9me3 signal at aging DAR loci for *L1cam* and *Efnb2* in CA1, visualized using IGV (version 2.15.4, https://igv.org/doc/desktop/). Separate tracks are shown for young (3 months) and aged (18 months) cells. BigWig files sourced from the Zhang dataset were loaded directly to IGV, allowing visualization without additional preprocessing of the original deposited dataset. The arrows on the left indicate the direction of enrichment of H3K9me3 at DAR-loci; an age−associated increase at *L1cam* and a decrease at *Efnb2*. The genomic region of aging DARs detected on these loci (aged-down DAR on L1cam, aged-up DAR on Efnb2), are shown on corresponding genome browser tracks.

**F**. H3K9me3 ChIP-qPCR analysis of *App*, comparing young and aged hippocampal cells. Data are normalized to aged samples (*p* = 0.0351; n = 4 biological replicates from different brains).

**G**. H3K27ac ChIP-qPCR analysis of *Psen1*, comparing young and aged hippocampal cells. Data are normalized to aged samples (*p* = 0.0351; n = 4 biological replicates from different brains).

Figure S6. **BACH2 activity reduced in aged DG neurons.**

**A–B**. Gene ontology enrichment analysis for biological processes in neuronal subtypes of TF motifs enriched on aged-down DARs (A) and aged-up DARs (B). The *x*–axis indicates the significance by −log_10_(*p*-value).

**C**. Violin plot of average expression scores for BACH2–putative target genes identified in Figure 5, in each cell of neuronal subtypes split by their age.

**D**. Violin plot of average gene expression for *Bach1* and AP-1 components, *Jund*, *Jun*, *Fos*, and *Fosl2* in DG neurons split by their age of sample collection.

**E**. Representative Western blot analysis of BACH2 and GAPDH in primary neurons transduced with AAV vectors expressing BACH2-GFP or control GFP.

**F**. Genome browser tracks displaying ATAC signals at Bach2 motif-containing genomic loci for *Camk2d,* following BACH2 overexpression in primary hippocampal neurons. Separate tracks are shown for each replicate (three for both control and Bach2 overexpression). The location of the Bach2 motif, identified in Figure 5E (Table S5), is indicated. N=3 biological replicates per group.

**G**. Violin plot of expression level (left) and motif enrichment (right) of Bach2 in human AD dataset. The plots were taken from <https://swaruplab.bio.uci.edu/singlenucleiAD/> (Morabito *et al*, 2021). The Kruskal–Wallis test was performed for significance (ns: non-significant, *p* > 0.05; *: *p* <= 0.05; **: *p* <= 0.01; ***: *p* <= 0.001, ****: *p* < 0.0001).
