# Supplementary material for: Age‐Associated Transcriptomic and Epigenetic Alterations in Mouse Hippocampus
Source: Aging Cell. 2025 Sep 28;24(11):e70233. doi: 10.1111/acel.70233 (PMC12608092; doi:10.1111/acel.70233)

Figure S1. Single-nuclei profiling of transcriptome and chromatin accessibility in the aging hippocampus of mice.

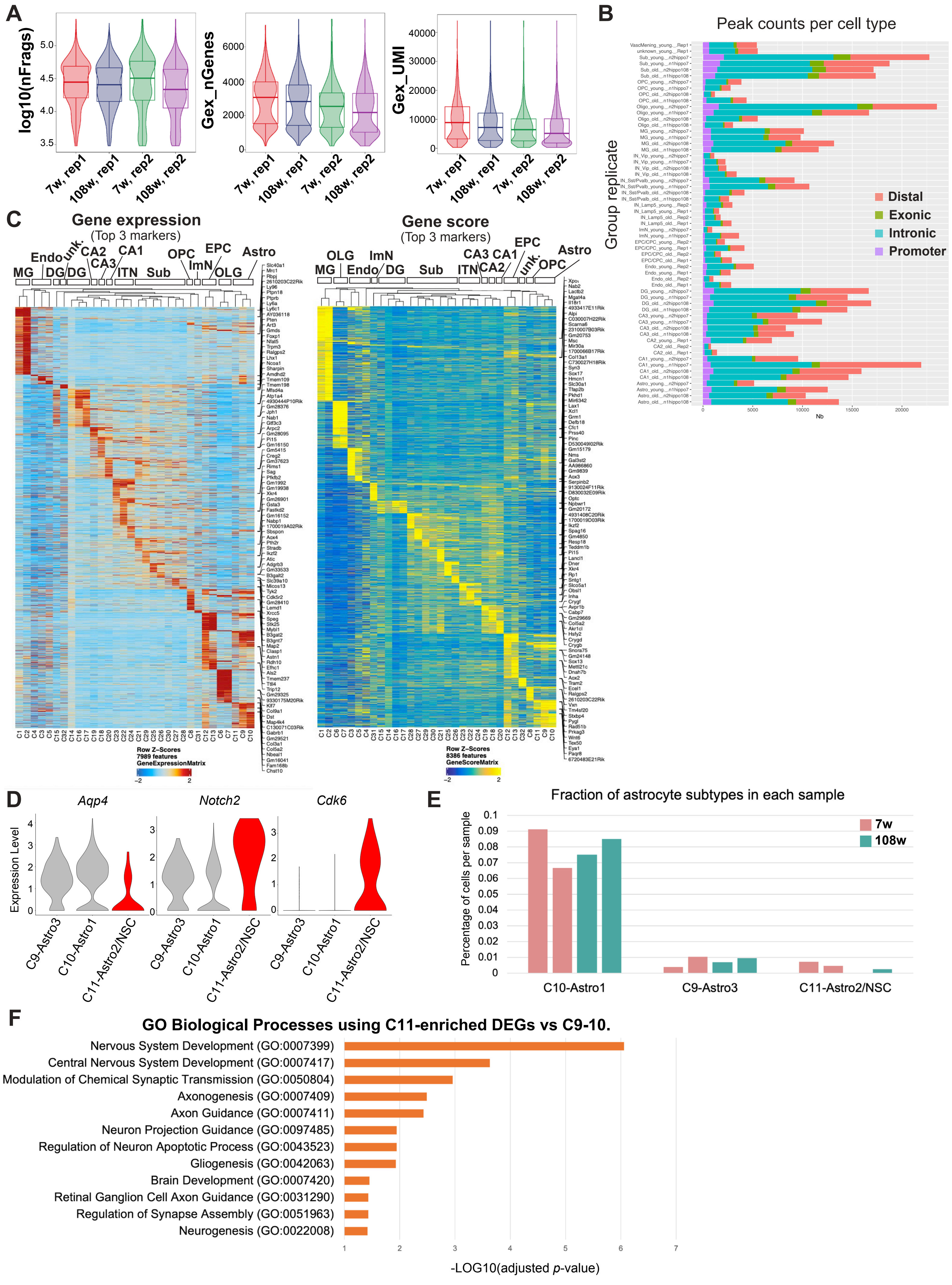

Figure S2. Quality control of clustering.

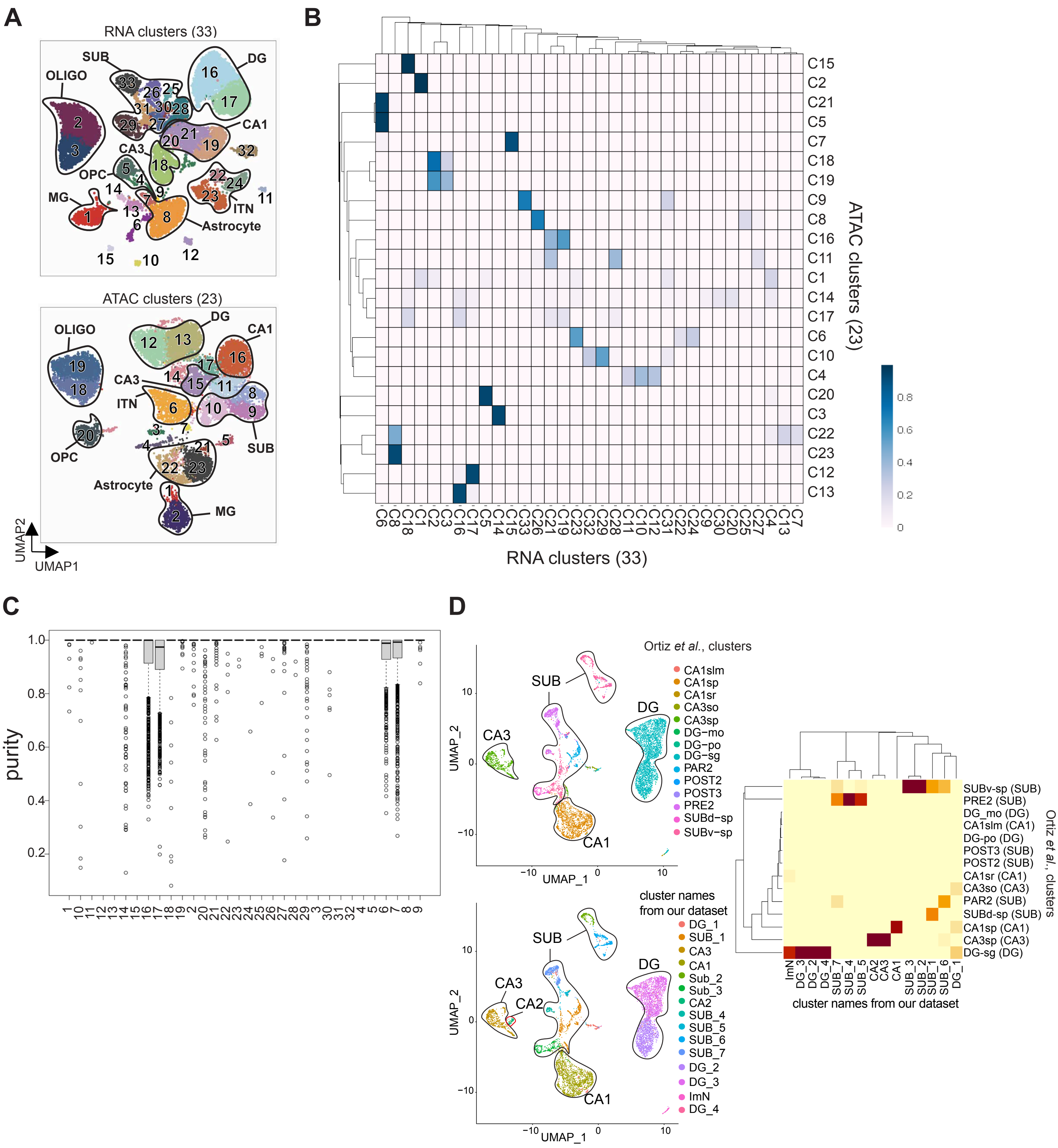

Figure S3. Cell type-specific dynamics of transcriptome and epigenome during hippocampal aging.

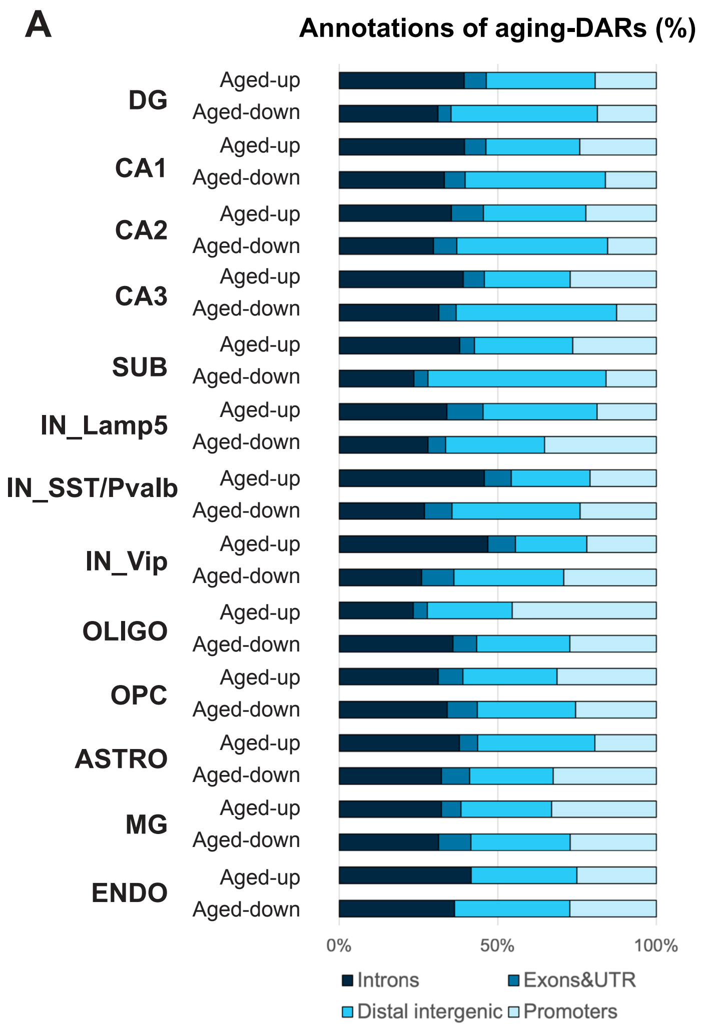

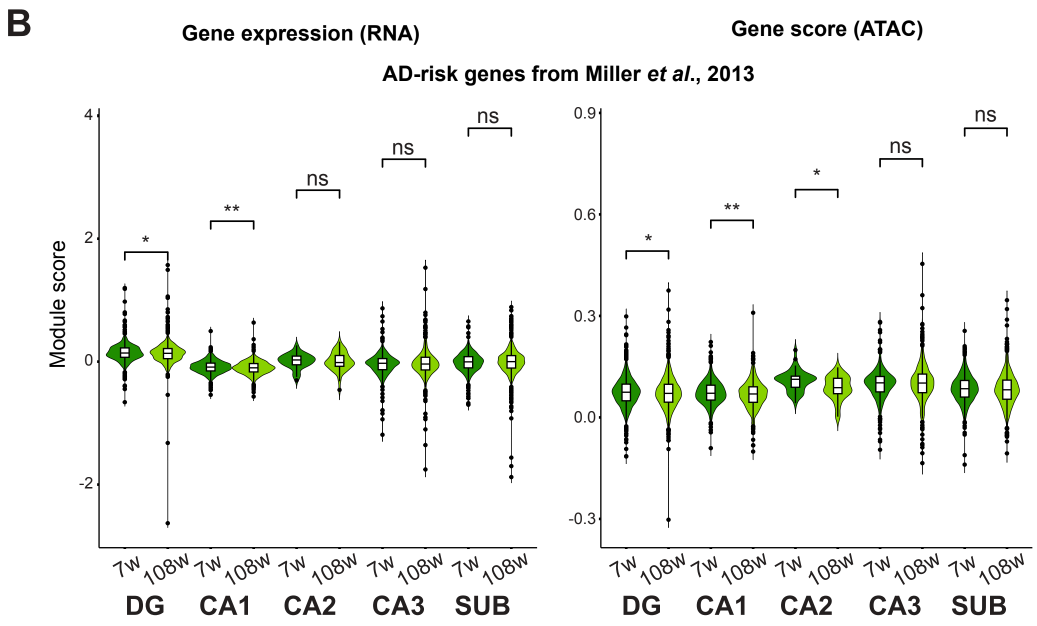

Figure S4. Dysregulation of neuronal genes in glial cells during hippocampal aging..

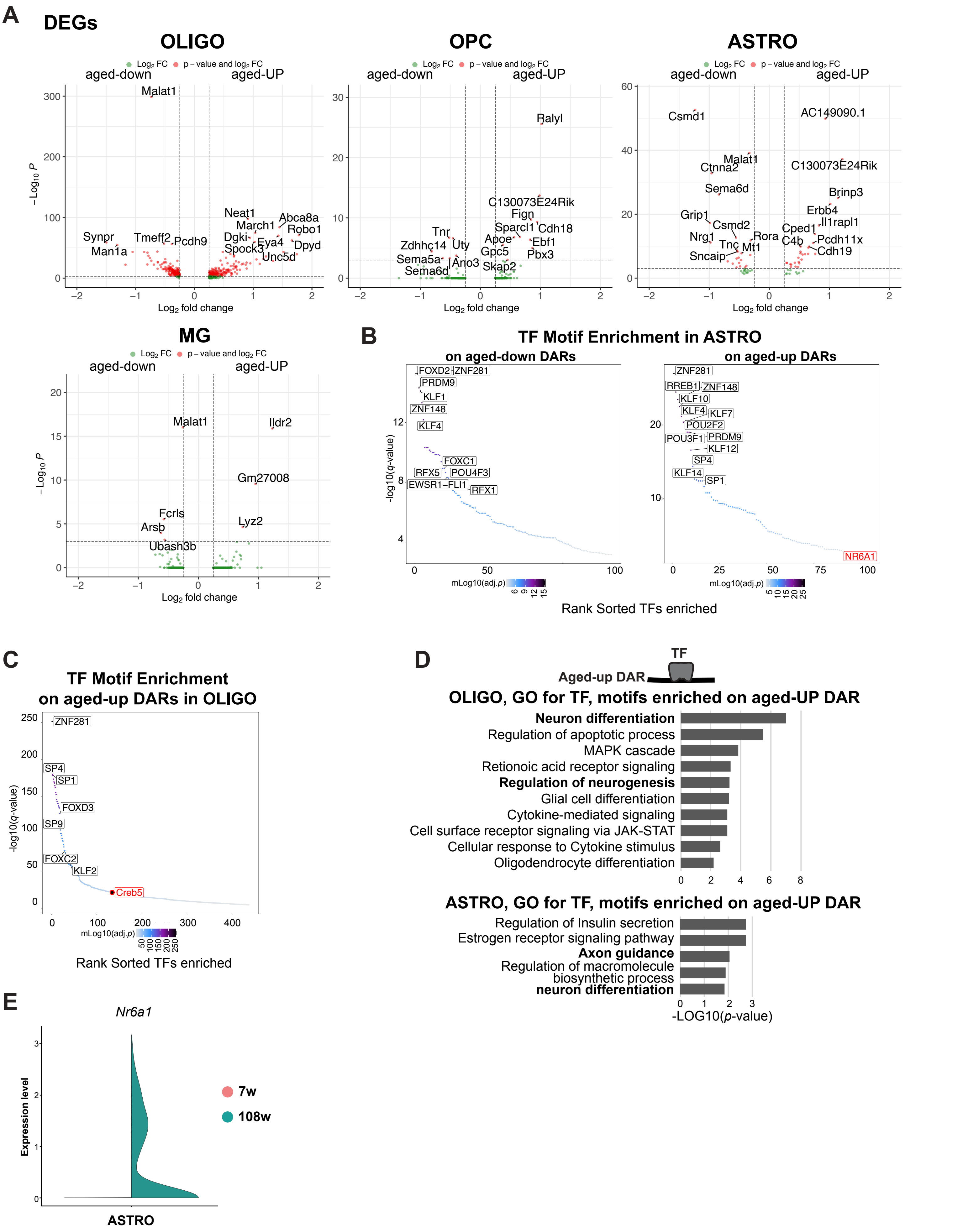

Figure S5. Chromatin accessibility-level dysregulations recapitulated aging features in neurons.

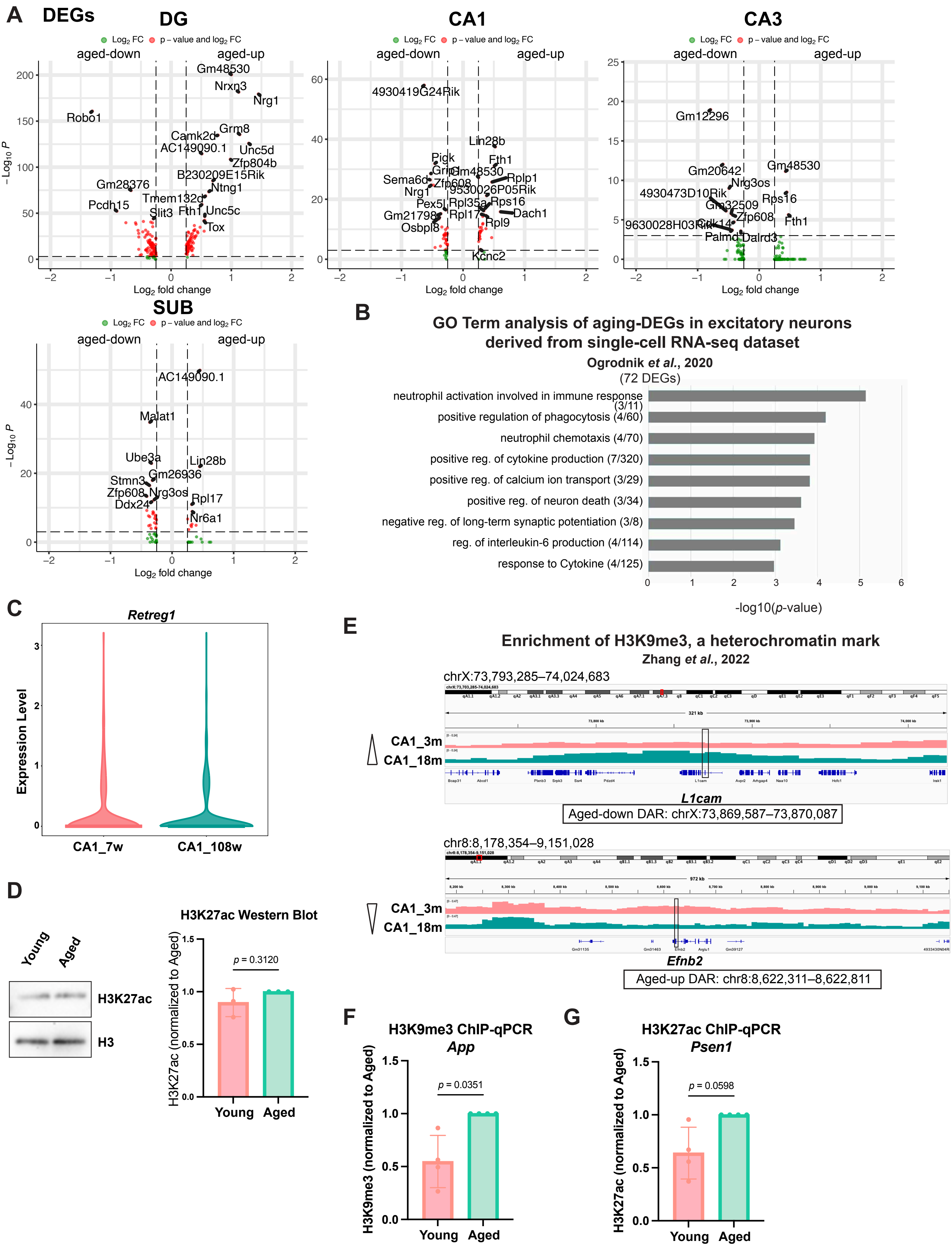

Figure S6. Aging-dependent transcriptomic and epigenetic changes in DG neurons.

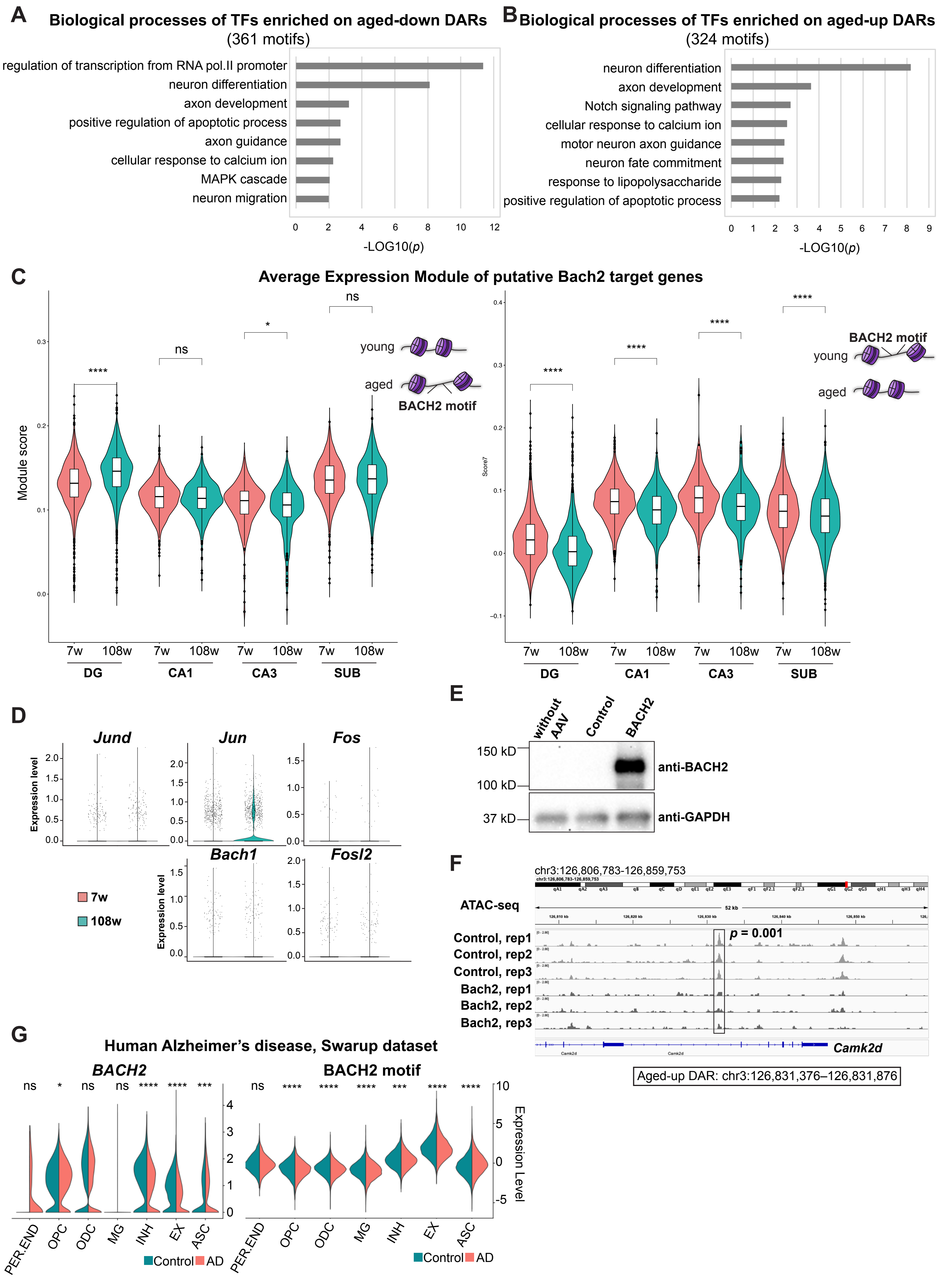

Supplement: Supplementary file 2 — Figure S1: Single‐nucleus profiling of transcriptome and chromatin accessibility in the mouse hippocampus with aging. (A) Quality‐control metrics of each replicate in 7‐week‐old and 108‐week‐old samples after filtering low‐quality cells (Methods). From left to right, violin plots of log10(fragment numbers), gene numbers, and UMI numbers for each cell are shown. Rep: replicate. (B) The number of peaks and their annotation are shown for each replicate of cell types from young or aged samples. (C) Heatmap of top three markers showing gene expression and gene activity in each cluster by hierarchical ordering. Major cell types are indicated in columns above. (D) Violin plot of normalized gene expression for representative markers of astrocyte (Aqp4) or NSC markers (Notch2, Cdk6) in astrocyte clusters of Figure 1B. (E) Fraction of astrocyte clusters in each sample. Y‐axis represents the percentage of cells per sample (Table S1). (F) Gene ontology enrichment analysis for biological processes in astrocytes using C11–enriched genes, derived from the DEG analysis comparing cluster 11 and clusters 9 and 10. The x‐axis indicates significance by −log10(adjusted p‐value). Figure S2: Quality control of clustering. (A) Visualization of cells on UMAP colored by RNA or ATAC clusters, with major cell types encircled according to their representative markers. (B) Heatmap of the confusion matrix representing the distribution of cells across clusters generated by RNA (rows) or ATAC (columns) modalities. Color scale represents the log10 (number of cells) for each RNA–ATAC cluster combination. (C) Boxplot of cluster purity for each cluster. The purity of neighborhood for each cell was computed, and cells were distributed according to their purity along the y‐axis; the median of each cluster is shown. (D) Visualization of cells colored by predicted cluster identities using Seurat's label transfer model with the Ortiz et al. dataset, and by cluster identities shown in Figure 1B. The heatmap [file ACEL-24-e70233-s003.pdf]
